# Microbial Translocation Does Not Drive Immune Activation in Ugandan Children Infected With HIV

**DOI:** 10.1093/infdis/jiy495

**Published:** 2018-08-11

**Authors:** Felicity C Fitzgerald, Edouard Lhomme, Kathryn Harris, Julia Kenny, Ronan Doyle, Cissy Kityo, Liam P Shaw, George Abongomera, Victor Musiime, Adrian Cook, Julianne R Brown, Anthony Brooks, Ellen Owen-Powell, Diana M Gibb, Andrew J Prendergast, A Sarah Walker, Rodolphe Thiebaut, Nigel Klein, The CHAPAS-3 Trial Team, Chifumbe Chintu, Veronica Mulenga, Desiree Kabamba, Dorothy Kavindele, Chishala Chabala, Musaku Mwenechanya, Monica Kapasa, Caroline C Zulu, Mox Kalumbi, Elias Chambula, Joyce Lungu, Marjory N Liusha, Dorothy Zangata, Dorica Masuka, Elias Chambula, Shadreck Chanshi, Terence Chipoya, Semy Zulu, Daniel Chola, Betty Chanda, Steven Malama, Chama Chama, Sylvia Mulambo, Mpala Mwanza, R Alice Asiimwe, J Vicent Tukei, Violet Korutaro, Justine Komunyena, Isaac Sebuliba, Muzamil Kisekka, Carolyn Nansubuga, N Justine Mpanga, Moses Matovu, Charles Okello, Sharon Kesande, Gladys Namutebi, E Glorius Tumuheirirwe, Immaculate Nagawa, Sarah Nakimera, Geoffrey Onen, Fatuma Kabasita, Fred Sunday, Dick Isabirye, Cissy Kityo, Victor Musiime, Grace Mirembe, Elizabeth Kaudha, Amos Drasiku, Bernard Bainomuhwezi, Priscilla Wavamunno, Florence Odongo, Constance Lukowe, Winnie Namala, Daniel Sseremba, Alison Balaba, Alice Kwaga, Joshua Kayiwa, Matthew Odera, Paul Oronon, Edith Bagurukira, Phyllis Mwesigwa, Philip Apugulu, Lincoln Mugarura, Eram David Williams, Denis Odoch, Immaculate Nankya, Emmanuel Ndashimyeeva, Eva Nabulime, James Abach, Willy Agings Odong, Beatrice Arach, Irene Claren Aciro, Joseph Omongin, Geoffrey Amone, Peter Okello, Philliam Aleti, Edward Otim, Patrick Kidega, Emmanuel Achol, Innocent Mwape, Joshua Zulu, Gabriel Chipili, Linda Chibesa, Diana M Gibb, A Sarah Walker, Margaret J Thomason, Adrian Cook, Ellen Owen-Powell, Alex Ferrier, David Baptiste, Charlotte Male, Brendan Murphy, Moira Spyer, Julia Kenny, Nigel Klein, David Burger, Quirine Fillekes, Angela Colbers, Helen McIlleron, Elwyn Chomba, Jose Ramos, Zainab Akol, Peter Elyanu, Harriet Nakimuli, Julia Kenny, Diana M Gibb

**Affiliations:** 1 Infection, Immunity, and Inflammation Programme; 2 University College London (UCL) Genomics, UCL Great Ormond Street (GOS) Institute of Child Health; 3 Microbiology, Virology, and Infection Prevention and Control, Camelia Botnar Laboratories, GOS National Health Service Foundation Trust; 4 Medical Research Council Clinical Trials Unit at UCL; 5 Blizard Institute, Queen Mary University of London, London, United Kingdom; 6 INSERM, Bordeaux Population Health Research Centre, UMR 1219, University of Bordeaux, ISPED; 7 Statistics in System Biology and Translational Medicine (SISTM Team), INRIA Research Centre; 8 Vaccine Research Institute (VRI), Créteil, France; 9 Joint Clinical Research Centre, Kampala; 10 Joint Clinical Research Centre, Gulu, Uganda; 11 Zvitambo Institute for Maternal and Child Health Research, Harare, Zimbabwe

**Keywords:** HIV, children, microbial translocation, immune activation, sequencing, pediatrics, Africa

## Abstract

**Objective:**

Immune activation is associated with morbidity and mortality during human immunodeficiency virus (HIV) infection, despite receipt of antiretroviral therapy (ART). We investigated whether microbial translocation drives immune activation in HIV-infected Ugandan children.

**Methods:**

Nineteen markers of immune activation and inflammation were measured over 96 weeks in HIV-infected Ugandan children in the CHAPAS-3 Trial and HIV-uninfected age-matched controls. Microbial translocation was assessed using molecular techniques, including next-generation sequencing.

**Results:**

Of 249 children included, 142 were infected with HIV; of these, 120 were ART naive, with a median age of 2.8 years (interquartile range [IQR], 1.7–4.0 years) and a median baseline CD4^+^ T-cell percentage of 20% (IQR, 14%–24%), and 22 were ART experienced, with a median age of 6.5 years (IQR, 5.9–9.2 years) and a median baseline CD4^+^ T-cell percentage of 35% (IQR, 31%–39%). The control group comprised 107 children without HIV infection. The median increase in the CD4^+^ T-cell percentage was 17 percentage points (IQR, 12–22 percentage points) at week 96 among ART-naive children, and the viral load was <100 copies/mL in 76% of ART-naive children and 91% of ART-experienced children. Immune activation decreased with ART use. Children could be divided on the basis of immune activation markers into the following 3 clusters: in cluster 1, the majority of children were HIV uninfected; cluster 2 comprised a mix of HIV-uninfected children and HIV-infected ART-naive or ART-experienced children; and in cluster 3, the majority were ART naive. Immune activation was low in cluster 1, decreased in cluster 3, and persisted in cluster 2. Blood microbial DNA levels were negative or very low across groups, with no difference between clusters except for Enterobacteriaceae organisms (the level was higher in cluster 1; *P* < .0001).

**Conclusion:**

Immune activation decreased with ART use, with marker clustering indicating different activation patterns according to HIV and ART status. Levels of bacterial DNA in blood were low regardless of HIV status, ART status, and immune activation status. Microbial translocation did not drive immune activation in this setting.

**Clinical Trials Registration:**

ISRCTN69078957.

## Teaser

We found no evidence of an association between microbial translocation and immune activation in Ugandan human immunodeficiency virus (HIV)–infected children over time during receipt of antiretroviral therapy or in comparison to HIV-uninfected controls. In this setting, other factors may be driving immune activation in both infected and uninfected children.

In 2016, 160000 children acquired human immunodeficiency virus (HIV) infection, approximately 90% of whom were in sub-Saharan Africa [1]. Untreated HIV infection results in chronic immune activation [2, 3] and associated poorer immune recovery, increased mortality, and increased morbidity [4, 5]. Immune activation influences CD4^+^ and CD8^+^ T cells, monocytes, and dendritic cells and is associated with increased expression of proinflammatory cytokines such as interleukin 1 (IL-1) and tumor necrosis factor (TNF) [2, 6], polyclonal B-cell activation, and hypergammaglobulinemia [7, 8]. Immune activation can persist, albeit at lower levels, despite viral suppression with antiretroviral therapy (ART) [2, 9]. Because immune activation pathways may differ with regard to drivers and impact, depending on setting and age group, quantifying HIV-related immune activation involves measuring levels of a broad range of cellular, soluble, and inflammatory markers (eg, T-cell activation, TNF, and C-reactive protein [CRP]) [10].

The causes of immune activation in children are poorly characterized. One potential driver is microbial translocation. It is postulated that depletion of intestinal CD4^+^ T cells during the early stage of HIV infection allows increased microbial translocation from the gut, driving a chronic immune response [11, 12]. These microbial products include immunostimulants such as lipopolysaccharide (LPS), a component of gram-negative bacterial outer membranes, and 16S ribosomal DNA (rDNA), which is common to all bacterial species. Increased levels of circulating LPS are associated with higher levels of immune activation in HIV-infected adults [4, 12]. Higher levels of LPS and 16S rDNA are associated with poorer immune restoration in patients receiving ART [11].

However, the evidence that microbial translocation drives immune activation is inconsistent, and quantification has proved challenging. 16S rDNA polymerase chain reaction (PCR) analysis can be contaminated by artifacts from endogenous/exogenous bacteria [13, 14], and without sequencing the PCR product it is impossible to exclude contamination. Some studies have not found an association between microbial translocation and immune activation [15, 16]. Other studies have been cross-sectional or retrospective [17–20]. Translocation may be due to, rather than a cause of, severe HIV disease [21]. There are few data from Africa. One large African longitudinal study (performed in Uganda) found no significant increase in microbial translocation during untreated disease progression in adults; a high CRP level was associated with mortality without a high LPS level [22, 23]. Data in children are limited and lack consistent associations, such as between 16S rDNA/LPS and T-cell activation or inflammatory markers [24–28].

Although microbial translocation is an attractive hypothesis, further longitudinal studies are needed, particularly among children in sub-Saharan Africa, since microbial translocation in HIV-infected children has not been consistently associated with cellular/soluble pathways of immune activation [24–27, 29]. We therefore investigated the impact of microbial translocation on cellular/soluble immune activation pathways and vascular damage in Ugandan HIV-infected children, compared with findings in age-matched, HIV-uninfected controls, using a panel of molecular techniques, including next-generation sequencing (NGS) of bacterial 16S rDNA in blood. We hypothesized that if microbial translocation drove immune activation in this cohort, we would detect significant differences in levels of plasma bacterial DNA between children with and those without immune activation.

## METHODS

### Study Population

We included HIV-infected children enrolled in Uganda in CHAPAS-3, a trial comparing the toxicity and efficacy of first-line ART drugs (abacavir, zidovudine, or stavudine, given with lamivudine and either nevirapine/efavirenz; clinical trials registration ISRCTN69078957) [30]. Eligible children were ART naive or were ART experienced and had been receiving a stable stavudine-containing first-line ART regimen for >2 years with a viral load (VL) of <50 copies/mL. Age, ART, weight, height, and comorbidities were recorded longitudinally. Hematologic parameters, biochemical parameters, and CD4^+^ T-cell counts were measured at weeks 6, 12, and 24 and every 24 weeks thereafter; VL was measured in stored samples at 48 and 96 weeks (Supplementary Materials). At baseline and week 12 visits, whole-blood specimens were collected and, within 2 hours, were separated into cell pellets and plasma by centrifugation at 1500×*g* for 15 minutes and stored at −80°C. For flow cytometry, whole-blood specimens were evaluated within 4 hours of collection.

The trial showed good clinical, virological, and immunological responses in all randomized groups, with a low level of toxicity [30]. For this substudy, we included all 120 ART-naive and 22 ART-experienced children recruited at the Joint Clinical Research Centre (Kampala, Uganda). A total of 143 HIV-uninfected controls were recruited for a single cross-sectional assessment from community well-child clinics (Kampala). These children had their HIV-uninfected status confirmed and were age matched (±1 year) at a ratio of 1:1 to enrolled HIV-infected children during the recruitment phase. After clinical assessment of each child, a 10-mL frozen plasma specimen was collected and stored without cell pellets [31]; 109 had a sufficient volume of plasma for evaluation by assays used in this study. For microbial translocation assays, these cross-sectional samples plus longitudinal samples collected from HIV-infected children at baseline (before ART initiation, for ART-naive children) and weeks 12 and 72 were used. For markers of immune activation, samples collected at weeks 0 and 96 were used.

### Microbial Translocation

Because broad-range 16S rDNA PCR analysis is vulnerable to contamination, we used a quantitative PCR (qPCR) panel targeting bacteria previously implicated in translocation (eg, Enterobacteriaceae species [32]), known gut bacteria (eg, *Bifidobacterium* species), and bacteria that could be gut derived or skin contaminants (eg, *Staphylococcus aureus*). Bacterial DNA was extracted from plasma and cell pellets (to detect phagocytosed bacteria); qPCR was performed to detect *Lactobacillus* species*, Bifidobacterium* species*, Fusobacterium* species, Enterobacteriaceae organisms*, S. aureus, Streptococcus pyogenes*, and *Staphylococcus* species, and then broad-range 16S rDNA PCR was performed, as previously described (Supplementary Materials) [33]. qPCR sensitivity varied between assays, from 0.1–1 to 1.3–13 colony-forming units (CFUs) per PCR run, using species-specific standards (Supplementary Table 1).

For broad-range 16S rDNA PCR, bacterial load (measured as the number of CFUs per PCR run) was quantified using standards of known concentrations (derived from *Escherichia coli*), and samples with equal or higher cycle thresholds than those of negative controls were considered to have negative results. Assay sensitivity was 5–50 CFUs per PCR reaction. CFUs and a ranking scheme of test results (ie, strong positive to negative), rather than copy numbers, were used because presentation of a single 16S gene copy number can lead to overinterpretation, owing to variance of such data even within species (Supplementary Materials). NGS was performed according to the Illumina protocol, with modifications for samples with a low biomass level (Supplementary Materials).

### Immune Activation and Inflammatory Markers

Because pathways between microbial translocation and markers of inflammation, cellular immune activation, and vascular damage are not well characterized in this population, we selected a broad range of markers. Coagulation factor III (Tissue Factor), and D-dimer levels were quantified using commercial enzyme-linked immunosorbent assay kits (R&D Systems, Technoclone) according to the manufacturers’ instructions. Levels of the following 17 biomarkers were assessed using MesoScale Discovery in accordance with the manufacturer’s protocols and were read using a QuickPlex SQ analyzer (MesoScale Discovery; Supplementary Table 2*A*) [31]: IL-1 receptor antagonist (IL-1RN), high-sensitivity CRP, TNF, interleukin 10, interleukin 6, the chemokines CXCL8 (IL-8) and CCL2 (also known as monocyte chemoattractant protein 1), angiopoietin 1, angiopoietin 2, E-selectin, P-selectin, the transmembrane glycoprotein ICAM-3, thrombomodulin, serum amyloid A, the glycoprotein SICAM-1, the cell adhesion molecule VCAM, and the growth factor VEGFA.

For cellular markers, 2 flow cytometry panels were used. First, the presence of HLA-DR^+^ and CD38^+^ was assessed in CD4^+^/CD8^+^ T-cell populations, to quantify activated (ie, double-positive) T cells [3]. Second, proliferation of CD4^+^ T cells was quantified using Ki67 in naive (CD45RA^+^), recent thymic emigrant (CD31^+^), and memory (CD45RA^–^CD31^–^) T cells. Flow cytometry was performed using the FACSCalibur platform (Becton-Dickinson; Supplementary Materials and Supplementary Table 2*B*) [31].

### Statistical Analysis

Baseline characteristics were compared between ART-naive and ART-experienced HIV-infected children and matched control groups, using the χ^2^ or Fisher exact test (for binary data) and the Mann-Whitney *U* test (for continuous data). Paired baseline and week-96 levels of cellular, inflammation, cardiovascular injury and thrombogenesis markers were compared for HIV-infected groups, using Wilcoxon signed rank tests. Associations between markers of immune activation and microbial translocation were assessed using Spearman rho correlation analysis (Supplementary Materials).

Given the large number of baseline and week-96 laboratory parameters (age-associated CD4^+^ and CD8^+^ T-cell counts, viral load, inflammation biomarker levels, and immune activation and proliferation marker levels), we identified clusters of children with more-similar phenotypes, using principal components analysis (based on the correlation matrix), followed by hierarchical clustering of the first 5 principal components by use of Ward’s distance. We used baseline data from HIV-uninfected controls for baseline and week-96 analyses, assuming minimal change over time. Microbial translocation markers measured before randomization and at week 72 were compared across clusters. Pelleted samples were only available from HIV-infected children and were therefore not used in clustering.

All statistical analyses were performed with R, version 3.2.1. No formal adjustment was made for multiple testing, but results were interpreted on the basis of the strength of associations.

### Ethical Considerations

The study was approved by UCL Research Ethics Committee (5019/001) and the Ugandan National Council for Science and Technology (HS1559). Written informed consent was obtained for storage and use of samples from caregivers, with participant assent obtained when appropriate.

## RESULTS

This study recruited 285 children: 142 HIV-infected children (120 were ART naive, and 22 were ART experienced) and 143 HIV-uninfected controls, of whom 109 had sufficient plasma specimens for all assays (89 were age matched to ART-naive children, and 20 were age matched to ART-experienced children; Supplementary Materials). Two controls (age matched to ART-naive children) were excluded because they were statistical outliers with high levels of immune activation (Supplementary Materials). HIV-infected children had low and comparable toxicity rates in each randomized arm [30]. There were 3 deaths (due to measles, measles/pneumonia, and Kaposi sarcoma), all among ART-naive children, after 25, 38, and 56 weeks of ART. Four ART-naive patients were lost to follow-up by week 72; 1 only had baseline samples, and the others were followed up for >24 weeks. Samples were available for microbial translocation/immune activation assays for most children at each time point (Supplementary Table 3).

At enrollment, the ART-experienced group had received ART for a median of 4 years (interquartile range [IQR], 2.6–4.3 years). ART-naive children were younger, had lower CD4^+^ T-cell counts and percentages, and lower levels of anthropometric markers at baseline and showed greater gains over time, compared with ART-experienced children (Table 1). The ART-naive group was approximately 6 months younger than controls; ART-experienced children and controls were similar in age. Both HIV-infected groups had lower CD4^+^ T-cell percentages than controls. The ART-naive group had lower anthropometric values than controls at baseline, whereas values were similar between the ART-experienced group and HIV-uninfected controls.

**Table 1.** Characteristics of Antiretroviral (ART)–Naive and ART-Experienced Human Immunodeficiency Virus (HIV)–Infected Children and Age-Matched HIV-Uninfected Controls

Characteristic	HIV-Infected Children			HIV-Uninfected Children			
	ART Naive (n = 120)	ART Experienced (n = 22)	*P*	Matched to Naive Group (n = 87)	*P* (vs ART-Naive Group)	Matched to ART- Experienced Group (n = 20)	*P* (vs ART- Experienced Group)
Male sex	59 (49)	10 (45)	.82	40 (46)	.67	12 (60)	.37
Age, y	2.8 (1.7–4.0)	6.5 (5.9–9.2)	<.001	3.3 (2.4–4.4)	.004	6.3 (5.7–8.9)	.89
Baseline CD4^+^ T-cell count, cells/mm^3^	922 (637–1451)	1188 (928–1813)	.02	1361 (1039–1728)	<.001	1010 (856–1343)	.11
Baseline CD4^+^ T-cell percentage	20 (14–24)	35 (31–39)	<.001	38 (34–43)	<.001	40 (35–45)	.06
Baseline viral load, copies/mL	386800 (166500– 1800000)	<100	NA	NA		NA	
Change in CD4^+^ T-cell count from baseline t o wk 96, cells/mm^3^	314 (−38–657)	−157 (−438–59)	<.001	NA		NA	
Change in CD4^+^ T-cell percentage from baseline to wk 96	17 (12–22)	3 (0–6)	<.001	NA		NA	
Viral load <100 copies/mL at wk 96	84/111 (76)	19/21 (91)	.16	NA		NA	
Baseline weight-for-age *z* score	−2.0 (−3.2 to −0.9)	−1.5 (−2.2 to −0.4)	.05	−0.6 (−1.2–0.1)	<.001	−1.0 (−2.1 to −0.1)	.57
Baseline height-for-age *z* score	−2.5 (−3.5 to −1.3)	−1.5 (−2.1 to −1.1)	.02	−0.8 (−2.1–0.1)	<.001	−1.3 (−2.5 to −0.3)	.52
Weight-for-age *z* score change from baseline to wk 96	1.0 (0.2–1.9)	−0.1 (−0.2–0.0)	<.001	NA		NA	
Height-for-age *z* score change from baseline to wk 96	0.8 (0.2–1.3)	−0.1 (−0.3 to −1.4)	<.001	NA		NA	

Data are no. or proportion (%) of children or median (interquartile range). Categorical variables were compared using the χ^2^ or Fisher exact test, and continuous variables were compared using the Mann-Whitney *U* test.

Abbreviation: NA, not applicable.

As expected, since ART-experienced children had virological suppression at enrollment, more ART-experienced children (91%; 95% confidence interval [CI], 70%–99%) than ART-naive children (76%; 95% CI, 67%–83%) had a VL of <100 copies/mL at week 96, but the difference was not significant (*P* = .16).

### Microbial Translocation

**Figure 1.**
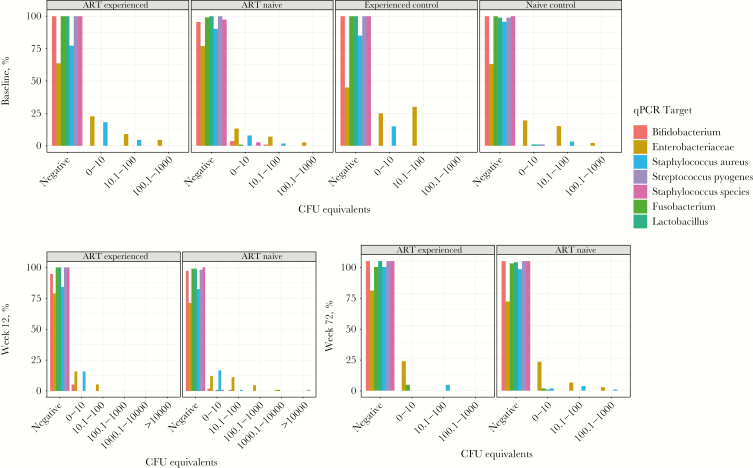
Quantitative polymerase chain reaction (qPCR) results over time ranked by negative to strong positive. Colony-forming unit (CFU) equivalents as compared to cycle threshold values of standards with a known CFU quantity and categorized from negative to strong positive. For each category, the total percentage of positive samples (ie, those with a result > 0) is equal to 100% minus the percentage of negative samples, shown at the left of each subpanel. ART, antiretroviral therapy.

Most plasma samples from all groups tested negative by qPCR (Figure 1), with the exception of Enterobacteriaceae organisms and *S. aureus*. Even in positive samples, levels were low. There was no significant difference between groups or over time in proportions of positive samples or in those testing weakly or strongly positive (*P* > .05; Supplementary Figure 1A and 1B), except in proportions positive for Enterobacteriaceae organisms between ART-naive and age-matched controls at baseline (23% [95% CI, 16%–32%] and 37% [95% CI, 27%–48%], respectively; *P* = .03). Because pellet samples were only available for HIV-infected children, comparisons to controls were not possible. The proportions positive for *S. aureus* and Enterobacteriaceae organisms were higher for pellets than plasma samples in both HIV-infected groups: among ART-naive children, 52% of pellets (95% CI, 42%–61%) and 10% of plasma specimens (95% CI, 5%–17%) were positive for *S. aureus*, and 69% (95% CI, 59%–77%) and 23% (95% CI, 15%–32%), respectively, were positive for Enterobacteriaceae species; among ART-experienced children, 64% of pellets (95% CI, 41%–83%) and 23% of plasma specimens (95% CI, 8%–45%) were positive for *S. aureus*, and 59% (95% CI, 36%–79%) and 36% (95% CI, 18%–62%), respectively, were positive for Enterobacteriaceae species.

Results of broad-range 16S rDNA PCR of plasma samples were similar across groups and time points and revealed no difference between HIV-infected and HIV-uninfected children at baseline (median value, 100 CFU equivalents (IQR, 23–204) and 102 CFU equivalents (IQR, 46–148) among ART-naive children and their age-matched controls, respectively [*P* = .45], and 133 CFU equivalents (IQR, 77–185) and 104 CFU equivalents (IQR, 53–152) among ART-experienced children and their age-matched controls, respectively [*P* = .25]; Supplementary Figure 1*C*). Pellets had higher median bacterial loads than plasma samples (1190 CFU equivalents [IQR, 440–2290] vs 113 CFU equivalents [IQR, 33–200]; *P* < .0001), but bacterial loads in pellets were similar in ART-naive and ART-experienced children (*P* = .65).

**Figure 2.**
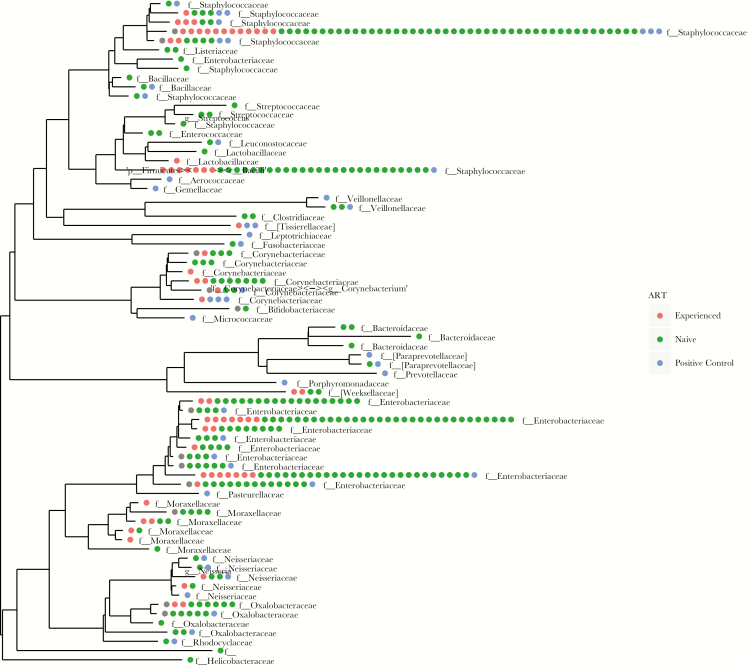
Results of next-generation sequencing. Positive controls are mock communities (Supplementary Materials). Phylogenetic tree, by antiretroviral therapy (ART) group (experienced or naive). Negative control operational taxonomic units (OTUs) were removed (rarefied), showing sparse OTUs, including Staphylococcaceae among other families. OTUs derived from both ART-experienced and ART-naive samples are distributed across the phylogenetic tree.

For NGS, 168 of 655 samples (26%; 105 of 140 pellets [75%] and 63 of 515 plasma samples [12%]) had sufficient quantities of the amplified 16S library to ensure successful sequencing. None of the 113 plasma samples from HIV-uninfected controls were sequenced successfully, as an insufficient quantity of the library was produced. After removing operational taxonomic units (OTUs) seen in negative experimental controls (Supplementary Materials), OTU numbers were sparse and dominated by Enterobacteriaceae organisms and staphylococci in both HIV-infected groups. Veillonellaceae, Clostridiaceae, Bacteroidaceae, and Bifidobacteriaceae organisms were also represented in low frequencies in both groups (Figure 2). Principal coordinates analysis (performed by the weighted Unifrac method [34]) revealed no clustering by ART-naive or ART-experienced status or by time point (Supplementary Figure 2).

### Immune Activation Markers

Levels of most cellular and humoral markers of immune activation, cardiovascular injury, and disordered thrombogenesis decreased significantly over time in the ART-naive group after ART initiation (Supplementary Table 4), except for D-dimer, angiopoietin 1 and 2, serum amyloid A, CXCL8, and IL-1RN (IL-1RA). For ART-experienced children, there were significant although less marked decreases in proliferating recent thymic emigrants (as measured by the CD45RA^+^CD31^+^Ki67^+^ cell percentage) and proliferating memory cells (as measured by the CD45RA^–^CD31^–^Ki67^+^ cell percentage), ICAM-3, interleukin 6, CCL2, VCAM, angiopoietin 2, and VEGFA. Compared with controls, all markers were higher in ART-naive children at baseline (*P* < .001), except for IL-1RN, angiopoietin 1, and coagulation factor III. Although ART-experienced children also had significantly higher levels of immune activation markers than controls in most assays (*P* < .0001), the differences were smaller. For several cardiovascular injury and disordered thrombogenesis markers (angiopoietin 1, E-selectin, ICAM-1, VEGFA, D-dimer, and thrombomodulin), there were no differences between ART-experienced children and controls with regard to levels of 3 inflammatory markers (CXCL8, serum amyloid A, and TNF) and markers of 1 cellular immune activation pathway (double-positive activated CD4^+^/CD8^+^ T cells). There was minimal correlation between immune activation and microbial translocation markers at early or late time points among HIV-infected ART-naive children and among HIV-infected ART-experienced children, and if any association was observed (eg, in the ART-experienced group, there was an association between 16S rDNA detection and the TNF level at week 96), it was inconsistent across time points (Supplementary Figure 3).

### Immune Activation Clusters

**Figure 3.**
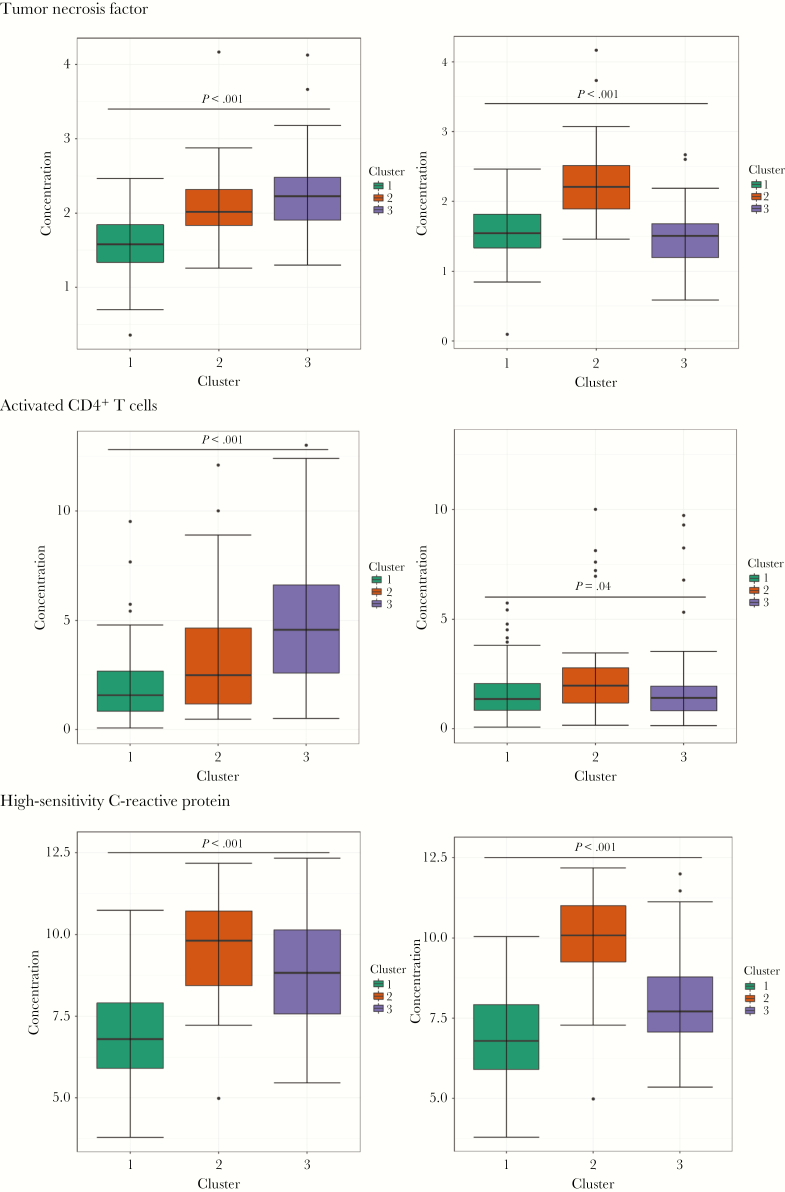
Box plots of log concentrations of tumor necrosis factor, activated CD4^+^ T cells (defined as, T cells double positive for HLA-DR and CD38), and high-sensitivity C-reactive protein, by cluster group, at baseline (week 0; left column) and week 96 (right column). *P* values were determined by the Kruskal-Wallis test. For concentrations of the complete set of biomarkers, see Supplementary Figure 5.

Based on values of the 19 markers of immune activation at enrollment (for all children) and week 96 (for HIV-infected children), children could be grouped into 3 distinct clusters that reflected different intrinsic phenotypes. Cluster 1 (n = 109) mostly comprised ART-experienced and HIV-uninfected children, cluster 2 (n = 33) included HIV-uninfected controls and children from both HIV-infected groups, and cluster 3 (n = 107) mostly included ART-naive children (Table 2 and Supplementary Figure 4). Cluster 1 had low levels of markers of immune activation at baseline and (for HIV-infected children) at week 96. The factors distinguishing cluster 2 were persistent immune activation at week 96, particularly in biomarkers of inflammation, cardiovascular injury, and disordered thrombogenesis, with less persistence of cellular markers (Supplementary Table 5, Figure 3, and Supplementary Figure 5). Cluster 3 had high levels of immune activation at week 0, which decreased by week 96 (Supplementary Figure 5). Of note, VL suppression at week 96 was less common in HIV-infected children in cluster 2 (3 of 13 [23%]) than those in cluster 3 (80 of 98 [83%]; *P* < .0001).

**Table 2.** Characteristics and Microbial Translocation Markers, by Cluster Group

Characteristic	Cluster 1 (n = 109)	Cluster 2 (n = 33)	Cluster 3 (n = 107)	*P*
Percentage of all children	44	13	43	
ART status at baseline, no. (%)				<.0001
Experienced	16 (15)	3 (9)	3 (3)	
Naive	6 (5)	10 (30)	104 (97)	
HIV uninfected	87 (80)	20 (61)	0 (0)	
Viral load among HIV-infected children				
Baseline	100 (100–245)	606275 (106390–1998000)	336520 (141390–949650)	<.0001
Wk 96	100 (100–100)	171 (110–61190)	100 (100–110)	<.0001
Virological suppression^a^ at wk 96				<.0001
Yes	20 (19)	3 (3)	80 (78)	
No	2^b^ (7)	9 (31)	18 (62)	
HIV uninfected	87 (81)	20 (19)	0 (0)	
Viral load data missing	0 (0)	1 (10)	9 (90)	
Bacterial data				
Baseline	109	33	107	
I-FABP level, pg/mL	118 (59 -222)	100 (57–165)	86 (40–138)	.008
16S rDNA positivity	99 (92)	28 (85)	84 (79)	.32
*Bifidobacterium* positivity	0 (0)	0 (0)	6 (6)	.02
*Staphylococcus aureus* positivity	9 (8)	4 (12)	10 (9)	.68
*Streptococcus pyogenes* positivity	0 (0)	1 (3)	0 (0)	.13
*Fusobacterium* positivity	1 (1)	0 (0)	0 (0)	.99
Enterobacteriaceae positivity	48 (44)	7 (21)	21 (20)	<.0001
*Staphylococcus* positivity	1 (1)	1 (3)	1 (1)	.51
*Lactobacillus* positivity	1 (1)	0 (0)	0 (0)	.99
Wk 72	109	33	106	
I-FABP level, pg/mL	150 (80–234)	159 (99–194)	146 (82–195)	.83
16S rDNA positivity	101 (93)	30 (91)	103 (96)	.18
*Bifidobacterium* positivity	0 (0)	0 (0)	0 (0)	
*Staphylococcus aureus* positivity	3 (3)	0 (0)	5 (5)	.26
*Streptococcus pyogenes* positivity	0 (0)	1 (3)	0 (0)	.13
*Fusobacterium* positivity	1 (1)	1 (3)	1 (1)	.51
Enterobacteriaceae positivity	48 (44)	3 (9)	31 (29)	.001
*Staphylococcus* positivity	0 (0)	0 (0)	0 (0)	
*Lactobacillus* positivity	0 (0)	0 (0)	1 (1)	.55

Data are no. (%) of children or median (interquartile range). Categorical variables were compared using the χ^2^ or Fisher exact test, and continuous variables were compared using the Mann-Whitney *U* test.

Abbreviations: HIV, human immunodeficiency virus; I-FABP, intestinal fatty acid binding protein; rDNA, ribosomal DNA.

^a^Defined as <100 copies/mL.

^b^Viral loads were 935 and 2140 copies/mL.

Markers of microbial translocation were low and similar across clusters, except for Enterobacteriaceae organisms: 48 of 109 children (44%) in cluster 1 were positive for these organisms, compared with 7 of 33 (21%) and 21 of 107 (20%) in clusters 2 and 3, respectively (*P* < .0001), a difference that remained at week 72 (Table 2). There was no evidence of differences across clusters in the proportions with positive results of the other microbial assays (*P* > .05).

## DISCUSSION

In this large, prospective study, with follow-up for >2 years and inclusion of HIV-uninfected controls, we aimed to evaluate the relationship between immune activation and microbial translocation in HIV-infected and uninfected children in Uganda. We used a wide range of cellular/soluble biomarkers to assess immune activation and comprehensively evaluated bloodstream bacterial DNA, using specific and broad-range PCR analyses alongside NGS. Plasma bacterial DNA was detected at very low levels, with minimal differences between HIV-infected and uninfected children or between ART groups or over time except for Enterobacteriaceae organisms, which were found in a higher proportion of controls than ART-experienced children at baseline. Where bacterial DNA was present, there was no association with levels of immune activation.

Immune activation decreased over time in most ART-naive children, and markers of immune activation tended to be lower in ART-experienced than ART-naive children, making them more similar to age-matched controls, as expected. Cluster analysis identified a group of ART-naive children (cluster 3) with immune activation decreasing over time along with viral load, but it also identified a group (cluster 2), comprising ART-naive, ART-experienced, and HIV-uninfected children, with high levels of immune activation. In both groups there was minimal association with microbial translocation. This suggests that, in a Ugandan setting, there may be causes of immune activation beyond either microbial translocation or HIV itself.

Techniques used for detecting microbial translocation are contentious. Previous studies have used LPS, soluble CD14 (a marker of monocyte activation), lipoteichoic acid (a component of gram-positive cell walls), lipopolysaccharide-binding protein (an acute-phase protein that binds LPS), and endotoxin core antibody (an antibody to LPS) as proxy markers of microbial translocation during HIV infection, with divergent, inconsistent results in comparison to clinical outcome, immune activation, and each other [11, 12, 16, 22, 24, 27–29, 35, 36]. Given the challenges in reproducibility and heterogeneity in previous studies [22, 29, 35, 37], we did not use LPS and instead focused on comprehensive evaluation of bacterial DNA. Broad-range 16S rDNA PCR has also shown conflicting results (Supplementary Materials). Even with sequencing, prior studies have not conclusively identified gut bacterial DNA in the bloodstream at levels greater than those of potential contaminants [11, 20, 32, 35]. By using a panel of molecular microbial techniques, including a modified broad-range 16S rDNA PCR assay used clinically for >15 years [13] and NGS, we combined sensitivity (from the qPCR assays) with specificity (from NGS) to identify bacterial species in the bloodstream that might have been derived from the gut.

The most consistently detected bacterial species across all groups was *S. aureus*, which could have been derived from the gut or a skin contaminant (despite efforts to minimize contamination). Without sequencing, it is difficult to extrapolate the degree to which previously demonstrated high frequencies of 16S rDNA positivity may have been due to skin contamination by *S. aureus* or other skin-colonizing bacteria, rather than to gut translocation. Using NGS, bacteria were identified that could be consistent with microbial translocation from the gut, such as *Veillonella* species and *Fusobacterium* species. However, they were found at very low levels in both ART-naive and ART-experienced children at baseline (Supplementary Figure 2).

Enterobacteriaceae organisms were found in HIV-infected individuals during 2 previous studies that used sequencing methods [20, 32]. Although in this study, Enterobacteriaceae species were detected using both qPCR and NGS, the frequency of detection by qPCR was consistently low over time (and lower than was considered clinically significant in previous use of the assay [38]) in both HIV-infected groups, indicating that the results may have been due to contamination. Enterobacteriaceae species are unlikely to be driving immune activation, because the ART-naive group experienced a diminution of immune activation over time and because positivity rates were higher in HIV-uninfected controls than cases at baseline (37% vs 23%; *P* = .03). Furthermore, given minimal differences between other groups, the assay’s inherent variability, and its vulnerability to contamination, these results may be false positives. However, the possibility remains that findings reflect levels of microbial translocation in both HIV-infected and uninfected groups that are low but not linked to immune activation. Given this possibility, despite previous provisos, the detection of Enterobacteriaceae organisms in more controls than HIV-infected children merits further investigation.

We were unable to sequence DNA in any plasma samples from HIV-uninfected controls, likely because of insufficient DNA present despite similar sample volumes, which may mean that there is a biologically significant difference in the quantities of bacterial DNA between HIV-infected and uninfected groups, as found in previous studies that used broad-range 16S rDNA PCR (without sequencing) [11, 20]. We did not have pellets available for controls, which in general yielded higher quantities of DNA for each assay, possibly because of detection of phagocytosed bacteria or due to the process of pelleting concentrating bacterial DNA. In future studies, pellets may be more useful in evaluating microbial translocation than plasma samples. However, it is important to emphasize that, even in pellets, despite lower immune activation, ART-experienced children had levels of bacterial DNA similar to those in ART-naive children. Future studies must consider the challenges associated with interstudy comparisons, as described in the Supplementary Materials.

From this comprehensive evaluation of bacterial DNA in the bloodstream of HIV-infected and HIV-uninfected children in Uganda, there is little evidence that microbial translocation is occurring at biologically significant levels or that it is driving immune activation, because markers of immune activation (along with viral load) fell significantly over time during ART but no difference was seen in levels of microbial DNA. This conclusion supports the finding that trials aiming to modify microbial translocation have not yet demonstrated clinically relevant improvements in HIV infection [39, 40]. Therefore, alternative modifiable causes of immune activation should be considered in this setting [9, 41], including HIV itself, via proteins such as Nef and gp120 [42, 43]; release from inhibition by regulatory mechanisms, such as the activity of T-regulatory cells [44]; other infections (both acute and chronic); and malnutrition. These may be more important than microbial translocation in driving immune activation.

Coinfections and malnutrition are of particular interest because children in cluster 2 had immune activation regardless of HIV/ART status. Acute and chronic infections significantly associated with immune activation during HIV infection include cytomegalovirus infection, malaria, tuberculosis, *Candida* infection, herpes zoster, and visceral leishmaniasis [45–49]. The association between these infections and immune activation in HIV-uninfected African children merits further investigation because there may be an opportunity to improve health outcomes beyond specifically targeting HIV. Malnutrition and environmental enteropathy may, independent of HIV, also drive immune activation [50].

Study limitations include the availability of baseline pellets only for HIV-infected children; these samples had higher yields of bacterial DNA and were more likely to be sequenced successfully by NGS. In future studies, the advantages of cell pellets over plasma specimens for bacterial NGS should be considered. Samples from only 1 time point were available for control children, so their results were used twice in the clustering analysis, based on the assumption that they had minimal changes over time. Acute infection was an exclusion criterion for control children, but some acute infections may have been missed. However, for HIV-infected children, the study was relatively large, including 142 children followed for 96 weeks.

In conclusion, based on comprehensive characterization of bacterial DNA in the bloodstream of HIV-infected children in Uganda as compared to controls from the same community, microbial translocation appeared to be low in HIV-infected children, regardless of receipt of ART and over time, whereas immune activation decreased over time in children commencing ART. A small but significant cluster of children had persistent immune activation regardless of HIV/ART status. Drivers of immune activation other than microbial translocation in both HIV-infected and HIV-uninfected children merit further investigation in African settings.

## Supplementary Data

Supplementary materials are available at *The Journal of Infectious Diseases* online. Consisting of data provided by the authors to benefit the reader, the posted materials are not copyedited and are the sole responsibility of the authors, so questions or comments should be addressed to the corresponding author.

Supplementary Material

## STUDY GROUP MEMBERS

Members of the CHAPAS-3 Trial Group are as follows: University Teaching Hospital, Lusaka, Zambia—Chifumbe Chintu, Veronica Mulenga, Desiree Kabamba, Dorothy Kavindele, Chishala Chabala, Musaku Mwenechanya, Monica Kapasa, Caroline C. Zulu, Mox Kalumbi, Elias Chambula, Joyce Lungu, Marjory N. Liusha, Dorothy Zangata, Dorica Masuka, Elias Chambula, Shadreck Chanshi, Terence Chipoya, Semy Zulu, Daniel Chola, Betty Chanda, Steven Malama, Chama Chama, Sylvia Mulambo, and Mpala Mwanza; Baylor Center of Excellence at Mulago Hospital, Kampala, Uganda—R. Alice Asiimwe, J. Vicent Tukei, Violet Korutaro, Justine Komunyena, Isaac Sebuliba, Muzamil Kisekka, Carolyn Nansubuga, N. Justine Mpanga, Moses Matovu, Charles Okello, Sharon Kesande, Gladys Namutebi, E. Glorius Tumuheirirwe, Immaculate Nagawa, Sarah Nakimera, Geoffrey Onen, Fatuma Kabasita, Fred Sunday, and Dick Isabirye; Joint Clinical Research Centre, Kampala—Cissy Kityo, Victor Musiime, Grace Mirembe, Elizabeth Kaudha, Amos Drasiku, Bernard Bainomuhwezi, Priscilla Wavamunno, Florence Odongo, Constance Lukowe, Winnie Namala, Daniel Sseremba, Alison Balaba, Alice Kwaga, Joshua Kayiwa, Matthew Odera, Paul Oronon, Edith Bagurukira, Phyllis Mwesigwa, Philip Apugulu, Lincoln Mugarura, Eram David Williams, Denis Odoch, Immaculate Nankya, Emmanuel Ndashimyeeva, and Eva Nabulime; Joint Clinical Research Centre, Gulu, Uganda—George Abongomera, James Abach, Willy Agings Odong, Beatrice Arach, Irene Claren Aciro, Joseph Omongin, Geoffrey Amone, Peter Okello, Philliam Aleti, Edward Otim, Patrick Kidega, and Emmanuel Achol; TASO Gulu—Gladys Aloyo and Robert Alani; Gulu Regional Referral Hospital—Alex Akera and Ciprian Odong; Centre for Infectious Disease Research in Zambia—Mpanji Siwingwa, Innocent Mwape, Joshua Zulu, Gabriel Chipili, and Linda Chibesa; MRC Clinical Trials Unit at UCL, London, United Kingdom—Diana M. Gibb, A. Sarah Walker, Margaret J. Thomason, Adrian Cook, Ellen Owen-Powell, Alex Ferrier, David Baptiste, Charlotte Male, Brendan Murphy, and Moira Spyer; Institute of Child Health, London—Julia Kenny and Nigel Klein; Radboud University Nijmegen Medical Center, Netherlands—David Burger, Quirine Fillekes, and Angela Colbers; University of Cape Town, South Africa—Helen McIlleron; Trial Steering Committee (independent members)—Elwyn Chomba (chair), Jose Ramos, Zainab Akol, Peter Elyanu, and Harriet Nakimuli (community); Data Monitoring Committee—Tim E. A. Peto (chair) and Margaret Siwale James Tumwine; and End Point Review Committee—Hermione Lyall (chair), Julia Kenny, and Diana M. Gibb.

## Notes

***Acknowledgments.***  We thank Dr Vania Toledo, for her unstinting administrative support; Lydia Nakiire, who performed flow cytometry; and all of the children, caregivers, and staff from the centers participating in the CHAPAS-3 trial. F. F., N. K., A. J. P., A. S. W., D. M. G., C. K., K. H., J. K., E. L., and R. T. were responsible for study concept and design. G. A., V. M., and E. O.-P. were responsible for sample collection. A. C. and E. O.-P. were responsible for data management. F. F., K. H., J. K., R. D., J. B., and A. B. were responsible for assay development and performance. F. F., E. L., J. K., R. D., L. S., A. S. W., R. T., and N. K. were responsible for data analysis. F. F. wrote the first manuscript draft, with further contributions from E. L., A. P., and A. S. W. All authors reviewed and approved the final draft.

***Disclaimer.***  The views expressed are those of the author(s) and not necessarily those of the National Health Service, the National Institute for Health Research, or the Department of Health, all of the United Kingdom.

***Financial support.*** This work was supported by the Medical Research Council (MRC; grant MR/K023535/1 to F. F., grant MC_UU_12023/26 to the MRC Clinical Trials Unit at University College London [UCL], and support to CHAPAS-3), the National Institute for Health Research Biomedical Research Centre at Great Ormond Street Hospital for Children National Health Service Foundation Trust (support to F. F.), UCL (support to F. F.), the Wellcome Trust (108065/Z/15/Z to A. J. P.), the European Developing Countries Clinical Trials Partnership (IP.2007.33011.006 to CHAPAS-3), the United Kingdom Department for International Development (support to CHAPAS-3), the Ministerio de Sanidady Consumo Spain (support to CHAPAS-3), and Cipla.

***Potential conflicts of interest.*** A. S. W. reports that her institution received funding from Janssen and Gilead Sciences outside of the submitted work. All other authors report no potential conflicts. All authors have submitted the ICMJE Form for Disclosure of Potential Conflicts of Interest. Conflicts that the editors consider relevant to the content of the manuscript have been disclosed.

## Notes

Presented in part: 2017 Conference on Retroviruses and Opportunistic Infections, Seattle, Washington, 13–16 February 2017. Abstract 0188.

## References

[1] Joint United Nations Programme on HIV/AIDS (UNAIDS). UNAIDS data 2017. Geneva: UNAIDS, 2017. 12349391

[2] Massanella M, Negredo E, Pérez-Alvarez N, et al CD4 T-cell hyperactivation and susceptibility to cell death determine poor CD4 T-cell recovery during suppressive HAART. AIDS 2010; 24:959–68. 20177358 10.1097/QAD.0b013e328337b957

[3] Giorgi JV, Hultin LE, McKeating JA, et al Shorter survival in advanced human immunodeficiency virus type 1 infection is more closely associated with T lymphocyte activation than with plasma virus burden or virus chemokine coreceptor usage. J Infect Dis 1999; 179:859–70. 10068581 10.1086/314660

[4] Ancuta P, Kamat A, Kunstman KJ, et al Microbial translocation is associated with increased monocyte activation and dementia in AIDS patients. PLoS One 2008; 3:e2516. 18575590 10.1371/journal.pone.0002516 PMC2424175

[5] Kuller LH, Tracy R, Belloso W, et al; INSIGHT SMART Study Group Inflammatory and coagulation biomarkers and mortality in patients with HIV infection. PLoS Med 2008; 5:e203. 18942885 10.1371/journal.pmed.0050203 PMC2570418

[6] Thieblemont N, Weiss L, Sadeghi HM, Estcourt C, Haeffner-Cavaillon N CD14lowCD16high: a cytokine-producing monocyte subset which expands during human immunodeficiency virus infection. Eur J Immunol 1995; 25:3418–24. 8566032 10.1002/eji.1830251232

[7] Weiss L, Haeffner-Cavaillon N, Laude M, Gilquin J, Kazatchkine MD HIV infection is associated with the spontaneous production of interleukin-1 (IL-1) in vivo and with an abnormal release of IL-1 alpha in vitro. AIDS 1989; 3:695–9. 2515876 10.1097/00002030-198911000-00002

[8] Lafeuillade A, Poizot-Martin I, Quilichini R, et al Increased interleukin-6 production is associated with disease progression in HIV infection. AIDS 1991; 5:1139–40. 1930778 10.1097/00002030-199109000-00014

[9] Klatt NR, Chomont N, Douek DC, Deeks SG Immune activation and HIV persistence: implications for curative approaches to HIV infection. Immunol Rev 2013; 254:326–42. 23772629 10.1111/imr.12065 PMC3694608

[10] McComsey GA, Kitch D, Sax PE, et al Associations of inflammatory markers with AIDS and non-AIDS clinical events after initiation of antiretroviral therapy: AIDS clinical trials group A5224s, a substudy of ACTG A5202. J Acquir Immune Defic Syndr 2014; 65:167–74. 24121755 10.1097/01.qai.0000437171.00504.41 PMC3943548

[11] Jiang W, Lederman MM, Hunt P, et al Plasma levels of bacterial DNA correlate with immune activation and the magnitude of immune restoration in persons with antiretroviral-treated HIV infection. J Infect Dis 2009; 199:1177–85. 19265479 10.1086/597476 PMC2728622

[12] Brenchley JM, Price DA, Schacker TW, et al Microbial translocation is a cause of systemic immune activation in chronic HIV infection. Nat Med 2006; 12:1365–71. 17115046 10.1038/nm1511

[13] Harris KA, Hartley JC Development of broad-range 16S rDNA PCR for use in the routine diagnostic clinical microbiology service. J Med Microbiol 2003; 52:685–91. 12867563 10.1099/jmm.0.05213-0

[14] Millar BC, Xu J, Moore JE Risk assessment models and contamination management: implications for broad-range ribosomal DNA PCR as a diagnostic tool in medical bacteriology. J Clin Microbiol 2002; 40:1575–80. 11980924 10.1128/JCM.40.5.1575-1580.2002 PMC130933

[15] Wittkop L, Bitard J, Lazaro E, et al; Groupe d’Epidémiologie Clinique du SIDA en Aquitaine Effect of cytomegalovirus-induced immune response, self antigen-induced immune response, and microbial translocation on chronic immune activation in successfully treated HIV type 1-infected patients: the ANRS CO3 Aquitaine Cohort. J Infect Dis 2013; 207:622–7. 23204178 10.1093/infdis/jis732

[16] Chevalier MF, Petitjean G, Dunyach-Rémy C, et al The Th17/Treg ratio, IL-1RA and sCD14 levels in primary HIV infection predict the T-cell activation set point in the absence of systemic microbial translocation. PLoS Pathog 2013; 9:e1003453. 23818854 10.1371/journal.ppat.1003453 PMC3688532

[17] Cassol E, Malfeld S, Mahasha P, et al Persistent microbial translocation and immune activation in HIV-1-infected South Africans receiving combination antiretroviral therapy. J Infect Dis 2010; 202:723–33. 20629534 10.1086/655229

[18] Nowroozalizadeh S, Månsson F, da Silva Z, et al Microbial translocation correlates with the severity of both HIV-1 and HIV-2 infections. J Infect Dis 2010; 201:1150–4. 20199244 10.1086/651430

[19] Lester RT, Yao XD, Ball TB, et al HIV-1 RNA dysregulates the natural TLR response to subclinical endotoxemia in Kenyan female sex-workers. PLoS One 2009; 4:e5644. 19461969 10.1371/journal.pone.0005644 PMC2680984

[20] Marchetti G, Bellistrì GM, Borghi E, et al Microbial translocation is associated with sustained failure in CD4+ T-cell reconstitution in HIV-infected patients on long-term highly active antiretroviral therapy. AIDS 2008; 22:2035–8. 18784466 10.1097/QAD.0b013e3283112d29

[21] Redd AD, Gray RH, Quinn TC Is microbial translocation a cause or consequence of HIV disease progression?J Infect Dis 2011; 203:744–5; author reply 746. 21220777 10.1093/infdis/jiq107 PMC3072727

[22] Redd AD, Dabitao D, Bream JH, et al Microbial translocation, the innate cytokine response, and HIV-1 disease progression in Africa. Proc Natl Acad Sci U S A 2009; 106:6718–23. 19357303 10.1073/pnas.0901983106 PMC2667149

[23] Redd AD, Eaton KP, Kong X, et al; Rakai Health Sciences Program C-reactive protein levels increase during HIV-1 disease progression in Rakai, Uganda, despite the absence of microbial translocation. J Acquir Immune Defic Syndr 2010; 54:556–9. 20463585 10.1097/QAI.0b013e3181e0cdea PMC2908216

[24] Wallet MA, Rodriguez CA, Yin L, et al Microbial translocation induces persistent macrophage activation unrelated to HIV-1 levels or T-cell activation following therapy. AIDS 2010; 24:1281–90. 20559035 10.1097/QAD.0b013e328339e228 PMC2888494

[25] Anselmi A, Vendrame D, Rampon O, Giaquinto C, Zanchetta M, De Rossi A Immune reconstitution in human immunodeficiency virus type 1-infected children with different virological responses to anti-retroviral therapy. Clin Exp Immunol 2007; 150:442–50. 17956580 10.1111/j.1365-2249.2007.03526.x PMC2219365

[26] Madrid L, Noguera-Julian A, Falcon-Neyra L, et al Microbial translocation and T cell activation are not associated in chronic HIV-infected children. AIDS 2014; 28:1989–92. 25259707 10.1097/QAD.0000000000000375

[27] Papasavvas E, Azzoni L, Foulkes A, et al Increased microbial translocation in ≤ 180 days old perinatally human immunodeficiency virus-positive infants as compared with human immunodeficiency virus-exposed uninfected infants of similar age. Pediatr Infect Dis J 2011; 30:877–82. 21552185 10.1097/INF.0b013e31821d141e PMC3173518

[28] Tincati C, Merlini E, Braidotti P, et al Impaired gut junctional complexes feature late-treated individuals with suboptimal CD4+ T-cell recovery upon virologically suppressive combination antiretroviral therapy. AIDS 2016; 30:991–1003. 27028142 10.1097/QAD.0000000000001015

[29] Pilakka-Kanthikeel S, Huang S, Fenton T, Borkowsky W, Cunningham CK, Pahwa S Increased gut microbial translocation in HIV-infected children persists in virologic responders and virologic failures after antiretroviral therapy. Pediatr Infect Dis J 2012; 31:583–91. 22333700 10.1097/INF.0b013e31824da0f5 PMC3648848

[30] Mulenga V, Musiime V, Kekitiinwa A, et al; CHAPAS-3 trial team Abacavir, zidovudine, or stavudine as paediatric tablets for African HIV-infected children (CHAPAS-3): an open-label, parallel-group, randomised controlled trial. Lancet Infect Dis 2016; 16:169–79. 26481928 10.1016/S1473-3099(15)00319-9 PMC4726762

[31] Kenny JM, Cook A, Mirembe G, et al Structural cardiovascular changes are reversible in HIV-infected children in Zambia and Uganda. In: Conference on Retroviruses and Opportunistic Infections Seattle, Washington.

[32] Merlini E, Bai F, Bellistrì GM, Tincati C, d’Arminio Monforte A, Marchetti G Evidence for polymicrobic flora translocating in peripheral blood of HIV-infected patients with poor immune response to antiretroviral therapy. PLoS One 2011; 6:e18580. 21494598 10.1371/journal.pone.0018580 PMC3073938

[33] Fitzgerald F, Harris K, Doyle R, Alber D, Klein N Short communication: Evidence that microbial translocation occurs in HIV-infected children in the United Kingdom. AIDS Res Hum Retroviruses 2013; 29:1589–93. 23972017 10.1089/aid.2013.0097 PMC3848482

[34] Lozupone C, Knight R UniFrac: a new phylogenetic method for comparing microbial communities. Appl Environ Microbiol 2005; 71:8228–35. 16332807 10.1128/AEM.71.12.8228-8235.2005 PMC1317376

[35] Sandler NG, Wand H, Roque A, et al; INSIGHT SMART Study Group Plasma levels of soluble CD14 independently predict mortality in HIV infection. J Infect Dis 2011; 203:780–90. 21252259 10.1093/infdis/jiq118 PMC3071127

[36] Dillon SM, Lee EJ, Kotter CV, et al An altered intestinal mucosal microbiome in HIV-1 infection is associated with mucosal and systemic immune activation and endotoxemia. Mucosal Immunol 2014; 7:983–94. 24399150 10.1038/mi.2013.116 PMC4062575

[37] Balagopal A, Gama L, Franco V, et al; ACTG A5175 Team. Serum inhibits detection of microbial translocation in HIV-1 and SIV infection: ACTG NWCS 319 [abstract 306]. In: 18th Conference on Retroviruses and Opportunistic Infections, Boston, Massachusetts, 2011.

[38] Tann CJ, Nkurunziza P, Nakakeeto M, et al Prevalence of bloodstream pathogens is higher in neonatal encephalopathy cases vs. controls using a novel panel of real-time PCR assays. PLoS One 2014; 9:e97259. 24836781 10.1371/journal.pone.0097259 PMC4023955

[39] Trois L, Cardoso EM, Miura E Use of probiotics in HIV-infected children: a randomized double-blind controlled study. J Trop Pediatr 2008; 54:19–24. 17878180 10.1093/tropej/fmm066

[40] Sandler NG, Zhang X, Bosch RJ, et al; AIDS Clinical Trials Group A5296 Team Sevelamer does not decrease lipopolysaccharide or soluble CD14 levels but decreases soluble tissue factor, low-density lipoprotein (LDL) cholesterol, and oxidized LDL cholesterol levels in individuals with untreated HIV infection. J Infect Dis 2014; 210:1549–54. 24864123 10.1093/infdis/jiu305 PMC4215074

[41] Deeks SG HIV infection, inflammation, immunosenescence, and aging. Annu Rev Med 2011; 62:141–55. 21090961 10.1146/annurev-med-042909-093756 PMC3759035

[42] Lee C, Liu QH, Tomkowicz B, Yi Y, Freedman BD, Collman RG Macrophage activation through CCR5- and CXCR4-mediated gp120-elicited signaling pathways. J Leukoc Biol 2003; 74:676–82. 12960231 10.1189/jlb.0503206

[43] El-Far M, Isabelle C, Chomont N, et al Down-regulation of CTLA-4 by HIV-1 Nef protein. PLoS One 2013; 8:e54295. 23372701 10.1371/journal.pone.0054295 PMC3553160

[44] Prendergast A, Prado JG, Kang YH, et al HIV-1 infection is characterized by profound depletion of CD161+ Th17 cells and gradual decline in regulatory T cells. AIDS 2010; 24:491–502. 20071976 10.1097/QAD.0b013e3283344895

[45] Casado JL, Abad-Fernández M, Moreno S, et al Visceral leishmaniasis as an independent cause of high immune activation, T-cell senescence, and lack of immune recovery in virologically suppressed HIV-1-coinfected patients. HIV Med 2015; 16:240–8. 25604328 10.1111/hiv.12206

[46] Eggena MP, Barugahare B, Okello M, et al T cell activation in HIV-seropositive Ugandans: differential associations with viral load, CD4+ T cell depletion, and coinfection. J Infect Dis 2005; 191:694–701. 15688282 10.1086/427516

[47] Maidji E, Somsouk M, Rivera JM, Hunt PW, Stoddart CA Replication of CMV in the gut of HIV-infected individuals and epithelial barrier dysfunction. PLoS Pathog 2017; 13:e1006202. 28241080 10.1371/journal.ppat.1006202 PMC5328284

[48] Lichtner M, Cicconi P, Vita S, et al CMV co-infection is associated with increased risk of Severe non-AIDS events in a large cohort of HIV-infected patients. J Infect Dis 2015; 211:178–86. 25081936 10.1093/infdis/jiu417

[49] Hsue PY, Hunt PW, Sinclair E, et al Increased carotid intima-media thickness in HIV patients is associated with increased cytomegalovirus-specific T-cell responses. AIDS 2006; 20:2275–83. 17117013 10.1097/QAD.0b013e3280108704

[50] Attia S, Versloot CJ, Voskuijl W, et al Mortality in children with complicated severe acute malnutrition is related to intestinal and systemic inflammation: an observational cohort study. Am J Clin Nutr 2016; 104:1441–9. 27655441 10.3945/ajcn.116.130518 PMC5081715

