# Supplementary material for: Microbial Translocation Does Not Drive Immune Activation in Ugandan Children Infected With HIV
Source: J Infect Dis. 2018 Aug 11;219(1):89–100. doi: 10.1093/infdis/jiy495 (PMC6284549; doi:10.1093/infdis/jiy495)
Supplement: Supplementary Material [file jiy495_suppl_supplementary_material.docx]

Appendix

Contents

Supplementary methods

Supplementary results

Appendix Table 1: Reaction constituents for qPCR reactions

Appendix Table 2A: Summary of biomarkers measured and immunophenotyping of CD4+/CD8+ cells.

Appendix Table 2B: Antibodies used in Flow cytometric analysis

Appendix Table 3: Sample availability for microbial translocation and immune activation assays at each time point.

Appendix Table 4: Changes in Immune Activation markers over time.

Appendix Table 5: Description of biomarkers that best characterize cluster 2 compared to other clusters defined by clustering analysis.

Appendix Figures 1A-1C: Changes in bacterial DNA detected over time by ART group.

Appendix Figure 2: Principal coordinates analysis after removal of negative control OTUs (rarefied), showing no clustering by sample type or timing.

Appendix Figure 3: Spearman correlations between markers of immune activation and microbial translocation (16S rDNA).

Appendix Figure 4: Subgroups of children identified from clustering of principal components.

Appendix Figure 5: Boxplots of biomarkers by cluster group at week 0 and week 96

References

**Supplementary methods**

*Sample collection*

Blood drawing was taken according to a standardised protocol involving wearing new gloves for each patient, using single use equipment including tourniquets and cleaning the skin beforehand with a sterile 70% isopropyl alcohol wipe and allowing it to air dry. Samples were collected in endotoxin-free tubes to minimise contamination.

*Viral Load*

Viral load was tested on stored samples using the Roche COBAS Ampliprep/Taqman version 2.0 and run with a 1/5 dilution (due to low sample volumes) of Basematrix 53, giving a lower limit of detection of 100 copies/ml.

*Flow cytometry*

For flow cytometry, whole blood was incubated with fluorochrome-conjugated antibodies to CD3, CD4, CD8, CD38 and HLA-DR to characterize T-cell activation; and CD4, CD45RA, CD31 and Ki67 to identify proliferating CD4+ T cell subpopulations. For activation, cells were washed with Automacs Buffer (Milteny Biotec Inc. Auburn, CA, USA) to remove unbound

antibody and lysed red blood cells and re-suspended in Phosphate buffered saline ((PBS), Invitrogen Ltd, UK) supplemented with 1% paraformaldehyde. For proliferation, red blood cells were lysed and washed with Automacs Buffer. The remaining cells were fixed then permeabilised with Perm Buffer containing Saponin (Sigma-Aldrich, UK).

Fluorochrome-conjugated antibodies are shown in Appendix Table 2B. Appropriate isotypic controls (mouse IgG1-PE and IgG2b-APC) were used to evaluate non-specific staining.

50,000 events were collected in the lymphocyte gate using morphological parameters (forward and side scatter). Data were processed using CellQuest Pro Software (Becton-Dickinson) and analysed using Flow Jo software version 7.6.4 (Treestar Inc).

*DNA extraction*

For extraction, DNA from pellet samples was extracted using the QIAamp DNA Mini Kit (Qiagen) with an additional bead-beating step. Plasma samples were extracted using an EZ1 robot and EZ1 Virus Mini Kit v2.0 with an additional bead-beating step. All samples were extracted on first thaw, and had an internal positive control (IPC, mouse genomic DNA, non-coding region) added prior to the bead-beating step. Negative extractions were included with each batch as a negative control.

*Quantitative PCRs*

All qPCRs were performed using 0.15-0.5uM of primer/probes, 2uL of molecular grade water (Bioline), 10μL of Quantifast Multiplex PCR+R master mix (Qiagen) and 5μL of sample (See Supplementary Appendix Table 1 for individual primer/probe sequences and concentrations). Assay sensitivity was 0.1-13 colony forming units (CFUs) per PCR reaction compared with species-specific standards of known concentration. All assays were performed on an Applied Biosystems 7500 Real-Time PCR System™ (Thermofisher) Fast protocol, with cycling conditions of 95°C for 5 minutes, then 45 cycles of 95°C (30s) and 60°C (30s). Pellets had significantly higher cycle thresholds for the IPCs than plasma samples (Two-tailed p value<0.0001) indicating template inhibition, so they were run at a 10-fold dilution.

*Broad range 16s rDNA PCR*

Broad range 16s rDNA PCR was carried out using of 5μL sample, 10.5μL PCR grade water (Bioline), 12.5μL Power SYBR Green master mix (Life Technologies) and 0.5μL 785F (5’ GGATTAGATACCCBRGTAGTC, 20pM) and 1175R (5’ ACGTCRTCCCCDCCTTCCTC, 20pM) amplifying the V5 – V7 region of the 16S rDNA gene[1]. Cycling conditions were 95°C for 10 minutes, 95°C for 15s, 60°C for 1 minute (40 cycles) and a dissociation cycle: 95°C for 15s, 60°C for 1 minute, 95°C for 15s.

*Next generation sequencing*

Next generation sequencing (NGS) library preparation used primers as above with individual 12 base pair (bp) error correcting barcodes/indices (different for forward and reverse to allow paired-end sequencing), and Illumina compatible adaptor, pad and linker sequences. Reagent components were: template DNA (5 μL), PCR buffer (Molzym®), dNTPs (25pmol/μL), forward and reverse primers (10 pmol/μL), Moltaq® enzyme (5 pmol/μL) and PCR grade water (Bioline®) to make reaction to 25 μL. Negative extractions, negative controls and mock communities were included. Microbial mock communities were obtained through BEI Resources, NIAID, NIH as part of the Human Microbiome Project: Genomic DNA from Microbial Mock Community A (Even, Low Concentration), v3.1, HM-278D, and Microbial Mock Community B (Staggered, Low Concentration), v5.2LHM-783D. Cycling conditions were 94°C for 3 minutes, 35 cycles of 94°C (30s), 60°C (40s), 72°C (90s), and final extension (72°C) before cooling. The resulting amplicon was cleaned and pooled in 96 wells using SequalPrep normalization plate kits (Invitrogen) and AMPure XP beads (Beckman Coulter) both as per manufacturer’s protocol. Primer dimer visualised using an Agilent high sensitivity DNA kit (hsDNA) an Agilent 2200 TapeStation Instrument was removed with repeated size selections using AMPure XP beads (Beckman Coulter). 2nM library quantified using a Qubit 2.0 (Life technologies) was loaded onto a MiSeq (Illumina) as per manufacturer’s protocol for 500 cycle V2 kits with the addition of custom sequencing primers using a 10% PhiX spike. The resulting FASTA files were analysed using QIIME[2]. Paired-end sequenced reads from each MiSeq run were merged using FLASH[3] and demultiplexed, pooled and assigned operational taxonomic units (OTUs) using QIIME v1.8.0[2]. OTUs were clustered at 97% similarity and assigned taxonomy against the Greengenes database (v.13.5)[4]. OTUs occurring at a count fraction of <0.001 were filtered out (minimum count fraction or MCF), as were OTUs seen in experimental negative controls, and a single random rarefaction carried out to produce an even sampling depth across all samples (1000 sequences per sample). OTUs seen in experimental negative controls were considered representative of unavoidable low-level background contamination and so removed from the analysis. Further analyses on both rarefied and unrarefied results were carried out in R Studio (v.0.99.896) using Phyloseq (v.1.10.0).

*Statistical Analysis*

To reduce the influence of outliers, values were truncated at the 2.5^th^ and 97.5^th^ percentiles. A UK reference population was used for anthropometric measurements as the WHO reference population does not include weights for the whole age range of the CHAPAS-3 cohort.[5, 6]

For cluster analysis, the 19 biomarker measurements were log_2_ transformed for normality; viral load was log_10_ transformed. CD4/CD8-for-age were calculated as the ratio of the subject’s CD4+/CD8+ T-cell count to those of healthy individuals of the same age[7, 8].

*Comment on limits of detection of broad range 16s rDNA PCR and units used*

In most previous studies, the quantification of 16s rDNA PCR has been recorded as 16S rDNA copies/μL as opposed to CFU equivalents/PCR reaction in comparison with standards of known CFUs, and a ranking scheme of negative to high positives (for the 16S rDNA PCR not for the qPCRs)[9-13]. A disadvantage of the former method is that as the 16S rRNA gene has variable copy numbers between or even within bacterial species, presenting the result as a single figure could lead to over-interpretation, especially in the absence of downstream sequencing to identify bacterial species present[14, 15].This means that differences of less than one log in copies/μL might be artefactual and due to the species of bacteria present as opposed to the quantities. Due to concern about the assay’s vulnerability to contamination, results were interpreted conservatively: the limit of detection was determined by the lowest CT value of negative extraction and negative control samples even if this was lower than the threshold indicated by the standards. This means the sensitivity (5-50 CFUs/PCR reaction) was lower than for specific qPCRs and so there may have been some false negative samples. Using cell pellet samples increased the yield of bacterial DNA and so is likely to prove more useful in future studies

In terms of external comparison, the sensitivity of the assay equates to a sensitivity of 80-800 copies/μL[16]. This is much lower than the sensitivity of 5 copies/μL quoted in Jiang *et al.* which is the method used in several subsequent papers[9-13]. The median levels in HIV infected individuals detected in these papers vary from 5-400 copies/μL, and in one study, adults with chronic HIV had levels between 500-9000 copies/μL[17]. With the exception of Chevalier *et al.,* the levels generated by the assay of Jiang *et al.* tend to be lower than in this study (converting from CFU equivalents to median copies/μL of 1600-4180), but it is important to note that straightforward comparisons are unlikely to be useful owing to variability in the assay and the contrasting quantification methods used. Also, the method described by Jiang *et al.* does not include sequencing, and uses 45 thermal cycles, more than used in this study, which might mean positive results are due to contamination[9]. An alternative method described by Kramski *et al.* uses a shrimp nuclease to decrease contaminant DNA prior to amplification[18]. This modified assay has a quoted sensitivity of 10 copies/reaction, and reported 500-2500 16S rRNA copies/mL in HIV-infected adults from Australia[18]. A further method, again without sequencing, used in a cohort of children in Spain found medians of 630 and 3162 copies/μL in HIV uninfected and infected children respectively[19, 20]. It can be seen there is considerable variation in levels generated by different assays, and in copy numbers detected even in using similar assays in different studies. In summary, considering the difference (or lack of difference) between groups within this study is likely to be more useful than external comparators, owing to different methods used. In the absence of an external assay calibrator or standard, the usefulness of interstudy comparison of quantitative data is limited, even when using pellet samples with higher bacterial yield.

**Supplementary Results**

Control matching was not exactly 1:1 as planned due to the large numbers of assays involved, so not all control children had sufficient plasma for all assays. In total 143 controls were recruited; 34 had insufficient plasma availability for all necessary assays leaving 109 controls for analysis. However two controls had extreme outliers for immune activation (e.g. CD4+CD45RA-CD31+Ki67+ 33% and 65% compared with median (IQR) of 11.6% (7.2-15.7) / 3.0% (1.6-5.9) in HIV-infected ART-naive and age-matched uninfected controls respectively) that did not fit within the three clusters. Their inclusion would not have impacted our conclusion hence they were excluded as statistical outliers.

**Appendix Table 1: Reaction constituents for qPCR reactions**

| Target | Reagent | Sequence | Sensitivity (CFU/ reaction) | Concentration/ Volume |
| --- | --- | --- | --- | --- |
| **Mix 1** | | | | |
| *Staphylococcus aureus* [21] | *coa-*F | 5’-GTAGATTGGGCAATTACATTTTGGAGG | 0.1-1 | 0.15uM |
|  | *coa-*R | 5’-CGCATCTGCTTTGTTATCCCATGTA |  | 0.15uM |
|  | *coa-*Probe | 5’FAM- TAGGCGCATTAGCAGTTGCATC-BHQ1 |  | 0.15uM |
| *Streptococcus pyogenes[22]* | *csrR-*F | 5’-TGGATGTGGTTGCAGGTTTAGAC | 0.1-1 | 0.3uM |
|  | *csrR-*R | 5’- CGGGCAAGTAGTTCTTCAATGG |  | 0.3uM |
|  | *csrR-*Pr | 5’-JOE- CGGTGCAGACGACTATATTGTTAAACC-BHQ1 |  | 0.2uM |
| IPC[22] | *Mus* | 5’-GGACACTATGCCCCTCCTTAGA |  | 0.1uM |
|  | *Mus* | 5’-AGCTCCAAACTCCGTCTCTGTAA |  | 0.1uM |
|  | *Mus* | 5’Cy5-TTGGGAACAAAACACCCATGGAAGGA-BHQ2 |  | 0.1uM |
| **Mix 2** |  |  |  |  |
| *Enterobacteriacae[22]* | F *dnaK* | 5’ ACCTGGGTACWACCAACTCTTGTGT | 1-10 | 0.25uM |
|  | R *dnaK* | 5’ GTCACTGCCTGACGTTTAGC |  | 0.25uM |
|  | Probe *dnaK* | 5’-JOE-AGGATGGTGAAACTCTGGTWGGTCAGCC-BHQ1* |  | 0.25uM |
| *Staphylococcus* species [22] | F *tuf* | 5’ CATTCCAACTCCAGAACGTGAYT | 1-10 | 0.1uM |
|  | R *tuf* | 5’-CACGACCAGTGATTGAGAATACG |  | 0.1uM |
|  | Probe *tuf* | 5’-CY5-TGAYAAACCATTCATGATGCCAGTTGAGG-BHQ2 |  | 0.1uM |
| **Mix 3** | | | | |
| *Fusobacterium spp.[23]* | F | 5’ GGATTTATTGGGCGTAAAGC | 0.5-5 | 0.1uM |
|  | R | 5’GGCATTCCTACAAATATCTACGAA |  | 0.1uM |
|  | Probe | JOE 5’CTCTACACTTGTAGTTCCG BHQ1 |  | 0.1uM |
| **Mix 4** | | | | |
| *Bifidobacterium spp.* | *xfp*-F | 5’ CAGCTBTCCGAGCACCAGAT | 0.5-5 | 0.5uM |
|  | *xfp-R* | 5’ TCCTTCGTWCACGTGATCG |  | 0.5uM |
|  | *xfp-Pr* | 5’ FAM- CTTCCTCGAGGWCTAC-MGB* |  | 0.5uM |
| **Mix 5** | | | | |
| *Lactobacillus spp.* | F | 5’ GCTGASGCHATGGAAA | 1.3-13 | 0.5uM |
|  | R (casei) | 5’ GTAGTTGAAGGTATGCAATTSG |  | 0.25uM |
|  | R (acidophilus) | 5’ GTTGTTGAAGGDATGCAATTSG |  | 0.25uM |
|  | Probe (casei) | 5’ FAM-TTGGSCASGATGGTG-MGB |  | 0.25uM |
|  | Probe (acidophilus) | 5’ FAM-TTGGTMACGATGGTG-MGB |  | 0.25uM |

**Appendix Table 2A Summary of biomarkers, methods and lower limit of detection per assay**

| Method | Biomarker | Units | Lower limit of detection |
| --- | --- | --- | --- |
| MSD | Thrombomodulin (TM) | ng/mL | 0.064 |
|  | Intracellular adhesion molecule 3 (ICAM-3) | ng/mL | 0.064 |
|  | Selectin-E (SEL-E) | ng/mL | 0.064 |
|  | Selectin-P (SEL-P) | ng/mL | 0.064 |
|  | Serum amyloid A (SAA) | ng/mL | 10.9 |
|  | C-reactive protein (CRP) | ng/mL | 1.33 |
|  | Soluble vascular cell adhesion molecule 1 (VCAM-1) | ng/mL | 6 |
|  | Intracellular adhesion molecule 1 (ICAM-1) | ng/mL | 1.03 |
|  | Interleukin-6 (IL-6) | pg/mL | 0.192 |
|  | CXCL8 (Interleukin-8, IL-8) | pg/mL | 0.132 |
|  | Interleukin-10 (IL-10) | pg/mL | 0.0806 |
|  | CCL2 (C-C motif chemokine receptor 2 /Monocyte chemoattractant protein -1) | pg/mL | 0.116 |
|  | Tumour necrosis factor (TNF) | pg/mL | 0.0798 |
|  | Vascular endothelial growth factor A (VEGFA) | pg/mL | 0.229 |
|  | Interleukin 1 receptor antagonist (IL-1RN/IL-1RA) | pg/mL | 2.44 |
|  | Angiopoietin 1 (ANGPT-1) | pg/mL | 24.4 |
|  | Angiopoietin 2 (ANGPT-2) | pg/mL | 2.44 |
| ELISA | Coagulation factor III (F3) (Tissue factor, TF) | pg/mL | 0.69 |
|  | D-dimer (DD) | ng/mL | 0 |

**Appendix Table 2B Antibodies used in Flow cytometric analysis**

| Human target molecule | Mouse isotype | Flurochrome Conjugate | Source |
| --- | --- | --- | --- |
| Activation panel | | | |
| HLA-DR | IgG2b | APC | BD Biosciences UK |
| CD3 | IgG | PerCP | BD Biosciences UK |
| CD8 | IgG1 | FITC | BD Biosciences UK |
| CD38 | IgG1 | PE | BD Biosciences UK |
| Proliferation panel | | | |
| CD4 | IgG1 | PerCP | BD Biosciences UK |
| CD45RA | IgG2b | APC | BD Biosciences UK |
| CD31 | IgG1 | PE | BD Biosciences UK |
| Ki67 | IgG | FITC | BD Biosciences UK |
| HLA-DR: Human leukocyte antigen-DR, CD: Cluster of differentiation, Ig: Immunoglobulin, FITC: fluorescein isothiocyanate, PerCP: peridinin chlorophyll protein, PE: phyoerythrin, APC: allophycocyanin. | | | |

**Appendix Table 3: Sample availability for microbial translocation and immune activation assays at each time point (excluding two naïve controls).**

|  | Microbial Translocation markers | | | | Immune activation markers | | | |
| --- | --- | --- | --- | --- | --- | --- | --- | --- |
|  | **Naïve** | **Experienced** | **Naïve Controls** | **Experienced Controls** | **Naïve** | **Experienced** | **Naïve Controls** | **Experienced Controls** |
|  |  |  |  |  |  |  |  |  |
| **Baseline** |  |  |  |  |  |  |  |  |
| Total children | 120 | 22 | 87 | 20 | 120 | 22 | 87 | 20 |
| Sample not available | 9 |  |  |  | 0 | 0 | 0 | 0 |
| Total samples available | 111 | 22 | 87 | 20 | 120 | 22 | 87 | 20 |
|  |  |  |  |  |  |  |  |  |
| **Week 12** |  |  | - | - |  |  |  |  |
| Died | 0 | 0 | - | - | - | - | - | - |
| Loss to follow up | 0 | 0 | - | - | - | - | - | - |
| Total in follow up at Week 12 | 120 | 22 | - | - | - | - | - | - |
| Sample not available | 11 | 3 | - | - | - | - | - | - |
| Total with samples available | 109 | 19 | - | - | - | - | - | - |
|  |  |  |  |  |  |  |  |  |
| **Week 72** |  |  |  |  |  |  |  |  |
| Died | 3 | 0 | - | - | - | - | - | - |
| Lost to follow up | 4 | 0 | - | - | - | - | - | - |
| Total in follow up at Week 72 | 113 | 22 | - | - | - | - | - | - |
| Sample not available | 2 | 1 | - | - | - | - | - | - |
| Total with samples available | 111 | 21 | - | - | - | - | - | - |
|  |  |  |  |  |  |  |  |  |
| **Week 96** |  |  |  |  |  |  |  |  |
| Died | - | - | - | - | 3 | 0 | - | - |
| Lost to follow up | - | - | - | - | 4 | 0 | - | - |
| Sample not available | - | - | - | - | 3 | 0 | - | - |
| Total samples available | - | - | - | - | 110 | 22 | - | - |

**Appendix table 4.** Cellular, inflammation, cardiovascular injury and disordered thrombogenesis markers over time respectively in the ART naive and ART experienced group, and their respective HIV uninfected control groups (Median, interquartile range)

| **Markers** | | **ART naive** | | | **ART experienced** | | | **HIV uninfected** | |
| --- | --- | --- | --- | --- | --- | --- | --- | --- | --- |
|  |  | Week 0 | Week 96 | P-value* | Week 0 | Week 96 | P-value* | ART naive controls | ART experienced controls |
| **Cellular markers (%)** | |  |  |  |  |  |  |  |  |
|  | CD8-HLA-DR+CD38- | 0.2 (0.1 - 0.5) | 0.2 (0.1 - 0.4) | **0.006** | 0.4 (0.2 - 0.9) | 0.2 (0.1 - 0.4) | **0.03** | 0.8 (0.1- 0.4) | 0.1 (0.1 - 0.2) |
|  | CD8-HLA-DR+CD38+ | 4.6 (2.4 – 8.0) | 1.2 (0.7 - 2.1) | **<0.0001** | 1.4 (0.7 - 1.87) | 1.2 (0.8 - 1.4) | 0.12 | 1.4 (0.8 - 2.4) | 2.0 (0.0 - 2.8) |
|  | CD8+HLA-DR+CD38- | 0.1 (0 - 0.5) | 0.1 (0.0 - 0.4) | 0.86 | 0.4 (0.1 - 0.7) | 0.1 (0.0 - 0.4) | 0.20 | 0.1 (0.0 - 0.4) | 0.2 (0.0 - 0.4) |
|  | CD8+HLA-DR+CD38+ | 11.5 (6.3 - 22.9) | 3.3 (2.2 - 6.0) | **<0.0001** | 3.1 (1.7 - 4.2) | 2.5 (1.0 - 4.4) | 0.18 | 4.0 (2.9 - 6.6) | 3.4 (1.9 - 3.9) |
|  | CD4+CD45RA-CD31+Ki67+ | 11.6 (7.2 - 15.7) | 3.1 (14 - 5.6) | **<0.0001** | 4.1 (2.3 - 6.1) | 2.8 (1.4 - 4.4) | 0.33 | 3.0 (1.6 - 5.9) | 2.6 (1.2 - 5.9) |
|  | CD4+CD45RA+CD31+Ki67+ | 1.2 (0.8 - 1.6) | 0.5 (0.3 - 0.7) | **<0.0001** | 1.0 (0.7 - 1.3) | 0.4 (0.3 - 0.6) | **<0.0001** | 0.6 (0.4 – 1.0) | 0.5 (0.3 - 0.9) |
|  | CD4+CD45RA-CD31-Ki67+ | 8.8 (5.3 - 13.1) | 3.2 (1.2 - 5.5) | **<0.0001** | 4.9 (2.2 - 6.4) | 2.6 (0.7 - 3.8) | **0.03** | 2.0 (0.6 - 4.1) | 1.1 (0.2 - 3.2) |
|  | CD4+CD45RA+CD31-Ki67+ | 0.9 (0 - 2.1) | 0.4 (0.2 - 0.7) | **<0.0001** | 0.7 (0.0 – 1.0) | 0.3 (0.1 - 0.3) | **0.03** | 0.4 (0.2 - 0.8) | 0.2 (0.0 - 0.7) |
| **Inflammation markers** | |  |  |  |  |  |  |  |  |
|  | ICAM-3 (ng/mL) | 15.0 (10.2 - 19.1) | 0.9 (0.7 - 1.2) | **<0.0001** | 6.3 (4.7 - 9.5) | 0.9 (0.6 - 1.2) | **<0.0001** | 1.3 (1.1 - 1.5) | 1.0 (0.8 - 1.4) |
|  | IL-6 (pg/mL) | 11 (6.5 - 16.6) | 0.8 (0.5 - 1.4) | **0.01** | 7.1 (5.3 - 12.5) | 0.9 (0.7 - 2.3) | **0.002** | 0.8 (0.5 - 1.3) | 1.2 (0.9 - 2.0) |
|  | CXCL8 (IL-8) (pg/mL) | 5.7 (2.4 - 11.2) | 3.6 (1.8 - 6.6) | 0.39 | 3.2 (1.6 - 6.8) | 3 (1.2 - 5.1) | 0.40 | 2.9 (1.8 - 4.5) | 2.8 (2.2 - 6.7) |
|  | IL-10 (pg/mL) | 11.5 (4.3 - 20.4) | 1.2 (0.8 - 2.3) | **<0.0001** | 8.1 (2.9 - 21.2) | 1.2 (0.5 - 2.6) | 0.16 | 1.7 (1.2 - 2.8) | 2.3 (0.6 - 4.1) |
|  | IL-1RN (IL-1RA) (pg/mL) | 368 (243 - 579) | 197 (146 - 340) | 0.42 | 137 (115 - 217) | 173 (112 - 283) | 0.30 | 332 (260 - 439) | 290 (205 - 367) |
|  | CRP (ng/mL) | 6,387 (1,889 -27,332) | 2,525 (822 – 11,955) | **0.002** | 2,873 (1,117 -4,708) | 2,118 (414 – 7,441) | 0.38 | 1,531 (341 – 4,935) | 733 (356 – 3,251) |
|  | Serum amyloid A (ng/mL) | 2,715 (934 – 15,748) | 2,179 (903 – 5,583) | 0.85 | 1,855 (620 – 6,708) | 1,387 (455 – 3,165) | 0.20 | 1,462 (398 – 7,003) | 1,237 (541 – 5,181) |
|  | TNF (pg/mL) | 9.3 (6.6 - 12) | 4.4 (2.9 - 5.7) | **<0.0001** | 3.5 (3.0 – 4.2) | 3.4 (2.6 - 4.5) | 0.12 | 5.8 (4.4 - 6.9) | 5.0 (3.8 - 6.4) |
| **Cardiovascular injury markers** | |  |  |  |  |  |  |  |  |
|  | Angiopoietin-1 (pg/mL) | 4,969(1,625 – 13,770) | 5,239 (1,895 -15,828) | 0.33 | 2,146 (324 -10,081) | 5,083 (736 -13,234) | 0.29 | 4,972 (1,925-9,719) | 2,529 (1,045-6,585) |
|  | Angiopoietin-2 (pg/mL) | 6,337 (4,233 – 8,957) | 6,320 (4,728 – 9,914) | 0.30 | 4,391 (3,937 – 6,127) | 7,111 (5,374 – 8,754) | **0.001** | 7,533 (5,660-9,996) | 6,764 (5,151-8,193) |
|  | Selectin-E (ng/mL) | 26 (14.9 - 38.1) | 12.6 (8.6 - 18.5) | **<0.0001** | 14 (12 - 22) | 11.4 (8.6 - 20.3) | 0.56 | 15.7 (9.7 - 23.6) | 13 (6 - 20) |
|  | ICAM-1 (ng/mL) | 1,487 (1,101 – 2,193) | 569 (456 - 805) | **<0.0001** | 923 (763 – 1,104) | 560 (413 - 698) | 0.11 | 795 (612 - 1307) | 702 (581 - 861) |
|  | CCL2 (MCP-1) (pg/mL) | 189 (137 - 257) | 66 (54 - 83) | **<0.0001** | 128 (117 - 190) | 64 (43 - 95) | **0.002** | 83 (65 - 103) | 78 (60 - 95) |
|  | Selectin-P (ng/mL) | 71 (42 - 105) | 42 (32 - 59) | **<0.0001** | 62 (55 - 93) | 48 (36 - 78) | 0.67 | 46 (29 - 65) | 24 (15 - 39) |
|  | VCAM (ng/mL) | 2,525 (1,915 – 3,553) | 574 (407 - 847) | **<0.0001** | 1,373 (1,047 – 1,957) | 518 (477 - 692) | **0.02** | 863 (636 – 1,565) | 767 (627 – 1,198) |
|  | VEGFA (pg/mL) | 213 (80 - 455) | 43 (22 - 84) | **<0.0001** | 51 (19 - 156) | 39 (17 - 61) | **0.03** | 58 (27 - 99) | 24 (16 - 58) |
| **Disordered thrombogenesis markers** | | |  |  |  |  |  |  |  |
|  | D-Dimer (ng/mL) | 79 (18 - 239) | 4.4 (3.2 - 52.7) | 0.08 | 5.9 (5.9 – 24.0) | 4.4 (0.2 - 17.1) | 0.45 | 2 (2 - 50) | 3 (2 - 61) |
|  | Thrombomodulin (ng/mL) | 5.5 (4.2 - 7.1) | 3.5 (2.6 - 4.9) | **<0.0001** | 4.7 (4.4 - 5.7) | 4.8 (3.4 - 5.8) | 0.58 | 4.6 (3.6 - 5.7) | 4.6 (3.4 - 6.1) |
|  | Coagulation Factor III (Tissue Factor) (pg/mL) | 42 (34 - 50) | 50 (37 - 61) | **0.04** | 56 (45 - 72) | 56 (32 - 65) | 0.81 | 45 (30 - 56) | 35 (10 - 46) |

*P-value: comparison between Week 0 and Week 96 by Wilcoxon signed rank test

**Appendix Table 5: Description of biomarkers that best characterize cluster-2 compared to other clusters**

|  | Biomarker type | Biomarker | Mean (sd) in cluster (log) | Overall mean (sd) (log) | P value cluster-2 vs others |
| --- | --- | --- | --- | --- | --- |
| Strong evidence that values are higher at Week 96 in cluster-2 | Inflammation | IL-6 | 1.12 (1.53) | 0.02 (0.96) | <0.0001 |
|  |  | TNF | 2.28 (0.60) | 1.61 (0.49) | <0.0001 |
|  |  | IL-1RN (IL-1RA) | 6.66 (0.87) | 5.75 (0.71) | <0.0001 |
|  |  | ICAM-3 | 0.45 (0.40) | 0.05 (0.42) | <0.0001 |
|  |  | IL-10 | 1.70 (1.23) | 0.67 (0.93) | <0.0001 |
|  |  | CRP | 9.89 (1.57) | 7.71 (1.77) | <0.0001 |
|  |  | Serum amyloid A | 10.24 (1.77) | 8.02 (1.79) | <0.0001 |
|  |  | CXCL8/IL-8 | 1.89 (1.33) | 1.42 (0.93) | 0.002 |
|  | Cardiovascular injury | ICAM-1 | 7.48 (0.50) | 6.63 (0.56) | <0.0001 |
|  |  | VCAM | 7.55 (0.52) | 6.69 (0.85) | <0.0001 |
|  |  | CCL2 (MCP-1) | 4.85 (0.58) | 4.35 (0.44) | <0.0001 |
|  |  | Selectin-E | 3.07 (0.50) | 2.69 (0.54) | <0.0001 |
|  |  | Angiopoietin-2 | 9.39 (0.57) | 8.92 (0.49) | <0.0001 |
|  |  | VEGFA | 4.41 (0.89) | 4.00 (0.87) | 0.004 |
|  | Disordered thrombogenesis | Thrombomodulin | 1.72 (0.37) | 1.44 (0.37) | <0.0001 |
|  |  | D-Dimer | 3.62 (2.29) | 2.23 (2.21) | 0.0001 |
|  | Cellular marker | CD4+CD45RA+CD31+Ki67+ | 2.97 (8.65) | 0.91 (3.37) | <0.0001 |
|  |  | CD3+CD8+HLA−DR+CD38+ | 7.64 (6.50) | 5.01 (4.50) | 0.0003 |
| Weak evidence of higher values at Week 96 in cluster-2 | Cardiovascular injury | Selectin-P | 4.01 (0.48) | 3.82 (0.48) | 0.02 |
|  | Cellular marker | CD4+CD45RA−CD31+Ki67+ | 6.53 (10.81) | 4.60 (5.26) | 0.02 |
| Weak evidence of lower values at Week 96 in cluster 2 | Disordered thrombogenesis | Coagulation factor III (Tissue Factor) | 3.67 (0.49) | 3.84 (0.45) | 0.02 |

1. V-test comparing mean in cluster-2 versus the other clusters.
2. CRP: high sensitivity C-reactive protein; CD: cluster of differentiation; CD45RA: an isoform of CD45; CXCL8: C-X-C motif chemokine ligand 8; HLA-DR: human leukocyte antigen D-related; ICAM: Intercellular Adhesion Molecule; Ki-67: a protein cellular marker of differentiation; IL: interleukin; IL-1RN (IL-1RA): Interleukin-1 Receptor Antagonist; CCL2 (MCP-1): C-C motif chemokine receptor 2; TNF: Tumour Necrosis Factor; VCAM: Vascular Cell Adhesion Molecule; VEGFA: Vascular Endothelial Growth Factor A

**Appendix Figure 1:** Changes in bacterial DNA detected over time by ART group. **Appendix Figure 1A** Change in proportions positive for *S.aureus* using qPCR assay (plasma) over time with 95% CIs by ART group.

**Appendix Figure 1B** Change in proportions positive for *Enterobacteriaceae* using qPCR assay (plasma) over time with 95% CIs by ART group

**Appendix Figure 1C** Change in bacterial load (median CFU equivalents compared with standards of known CFUs with IQRs) by broad-range 16S rDNA PCR (plasma) over time by ART group

**Appendix Figure 2** Principal coordinates analysis after removal of negative control OTUs (rarefied), showing no clustering by sample type or timing. ART naïve and ART experienced samples distributed throughout.

**Appendix Figure 3. Spearman correlations between markers of immune activation and microbial translocation (16S rDNA).**

A. markers of immune activation (week 0) and microbial translocation (trial week -2)

B. markers of immune activation (week 96) and microbial translocation (week 72)


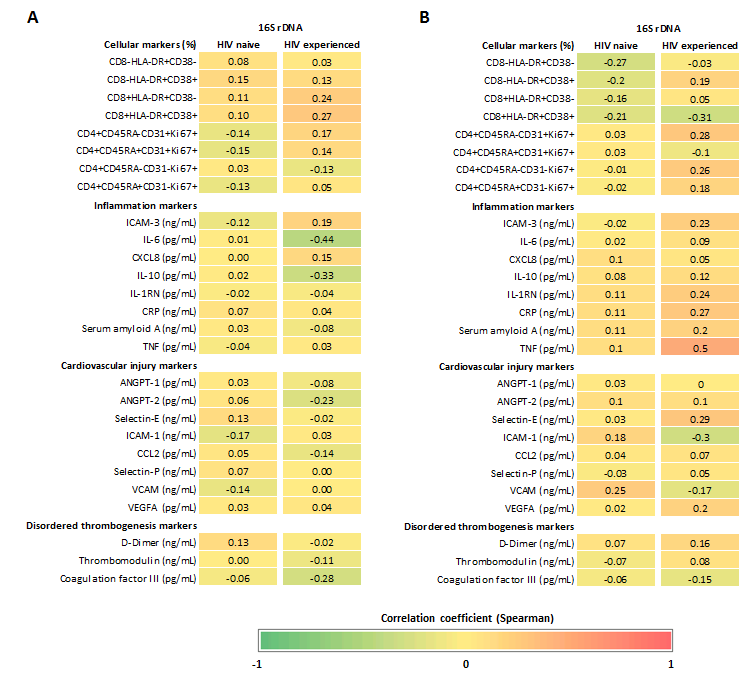


**Appendix Figure 4:** Subgroups of children identified from clustering of principal components.

A: Factor Map describing the three clusters on the first two principal components.

B: Clustering distribution by HIV/ART status at baseline

C: Distribution of clustering by viral suppression status at week 96


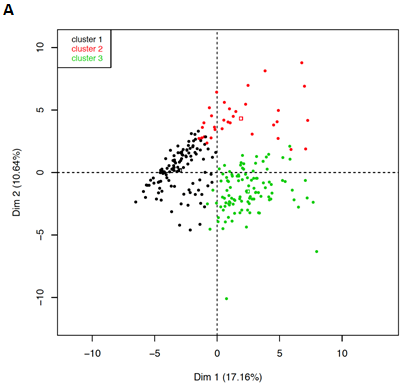


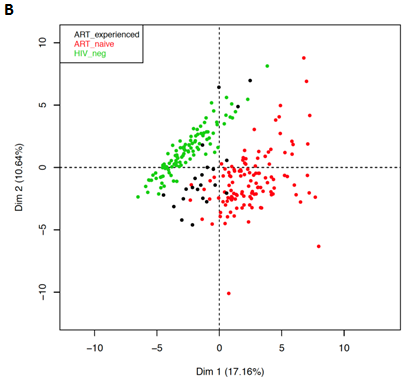

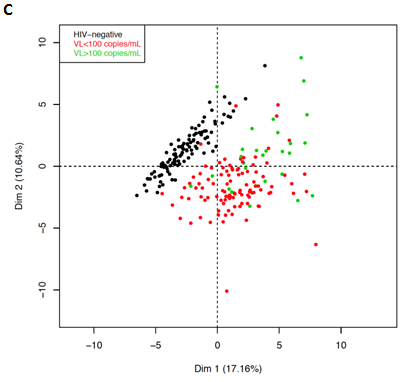


Parameters used for cluster analysis: CD4-for-age; CD8-for-age; viral load; cellular markers (); cellular markers (CD8-HLA-DR+CD38-, CD8-HLA-DR+CD38+, CD8+HLA-DR+CD38-, CD8+HLA-DR+CD38+, CD4+CD45RA-CD31+Ki67+, CD4+CD45RA+CD31+Ki67+, CD4+CD45RA-CD31-Ki67+, CD4+CD45RA+CD31-Ki67+); inflammation markers (ICAM-3, IL-6, CXCL8, IL-10, IL-1RN, CRP, Serum amyloid A, TNF); cardiovascular injury markers (ANGPT-1, ANGPT-2, Selectin-E, ICAM-1, CCL2, Selectin-P, VCAM, VEGFA); disordered thrombogenesis markers (D-Dimer, thrombomodulin, coagulation factor III) measured at week 0 and week 96.

**Appendix Figure 5: Panel boxplots of biomarkers by cluster group including both baseline (week 0: W0) and week 96 (W96) timepoints. Logged values used.**

CRP: high sensitivity C-reactive protein; CD: cluster of differentiation; CD45RA: an isoform of CD45; HLA-DR: human leukocyte antigen D-related; ICAM: Intercellular Adhesion Molecule; Ki-67: protein cellular marker of differentiation; IL: interleukin; IL-1RN: Interleukin-1 Receptor Antagonist; CCL2: C-C motif chemokine receptor 2 (MCP-1); TNF: Tumour Necrosis Factor; VCAM: Vascular Cell Adhesion Molecule; VEGFA: Vascular Endothelial Growth Factor A.

Cluster 1 is denoted in green, cluster 2 in orange and cluster 3 in purple.

**HIV related variables** **
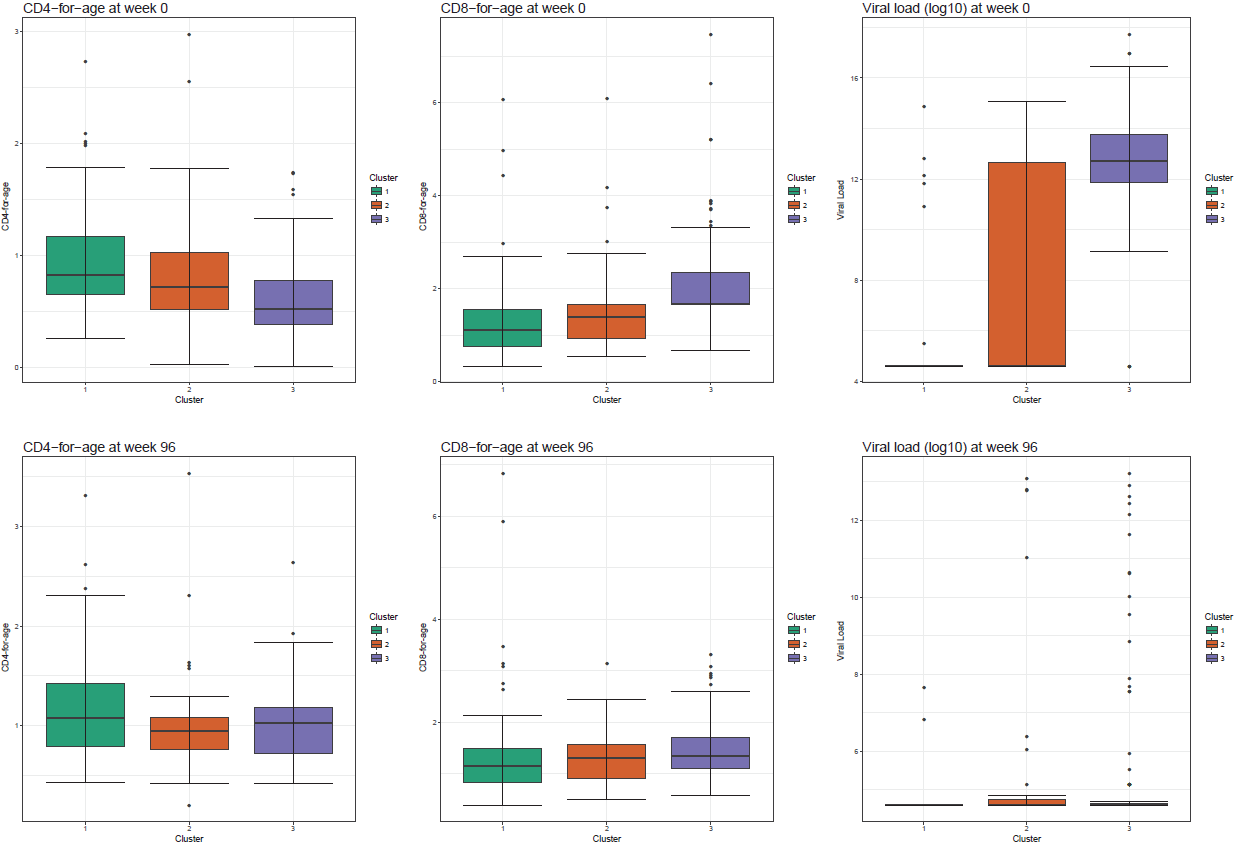
**

**Cellular markers**

**
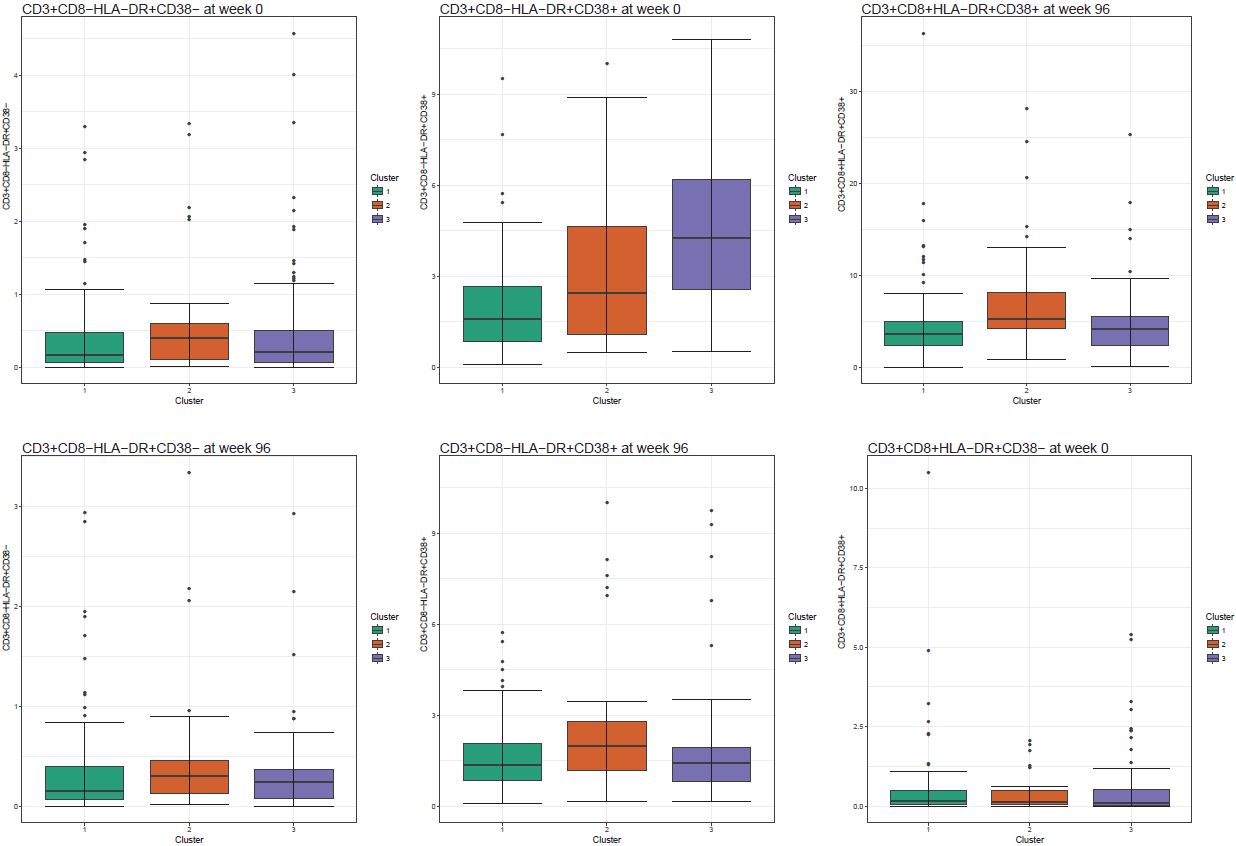
**


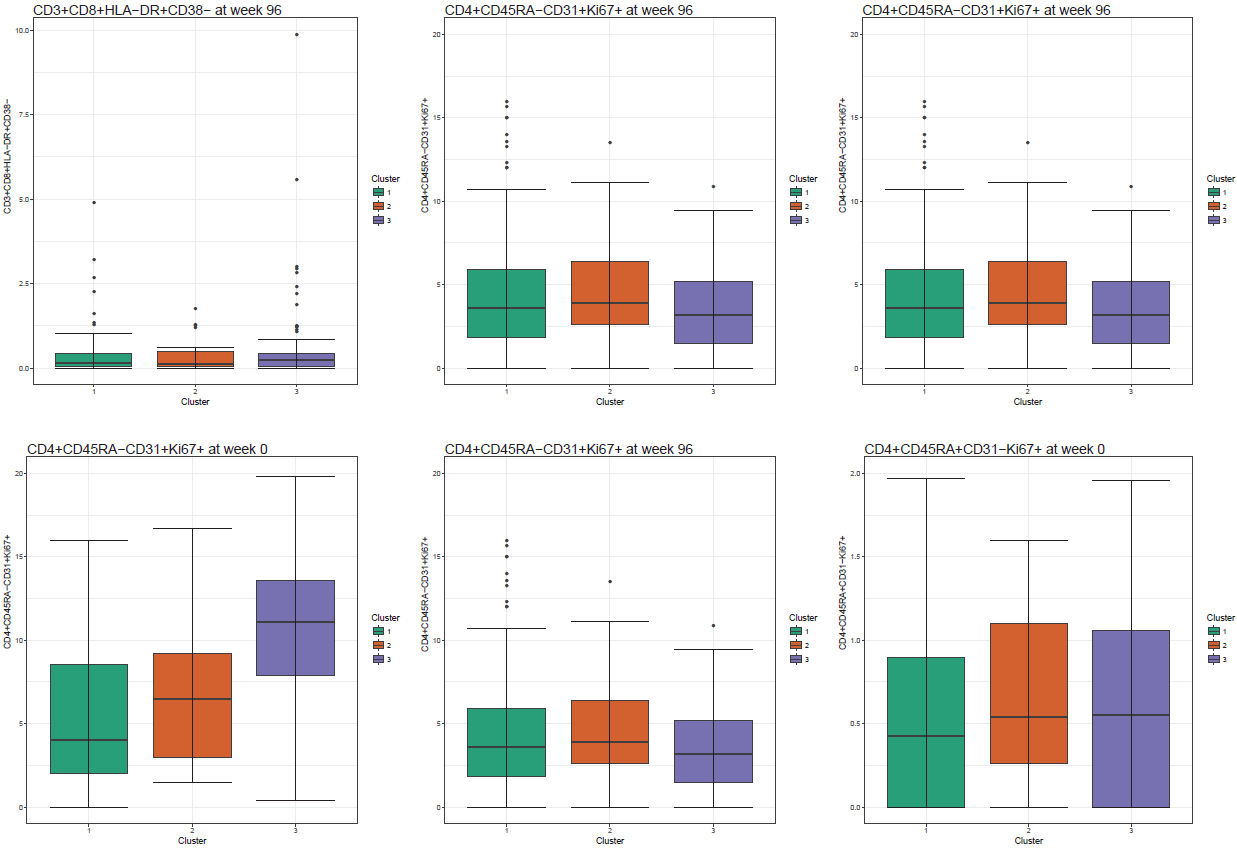


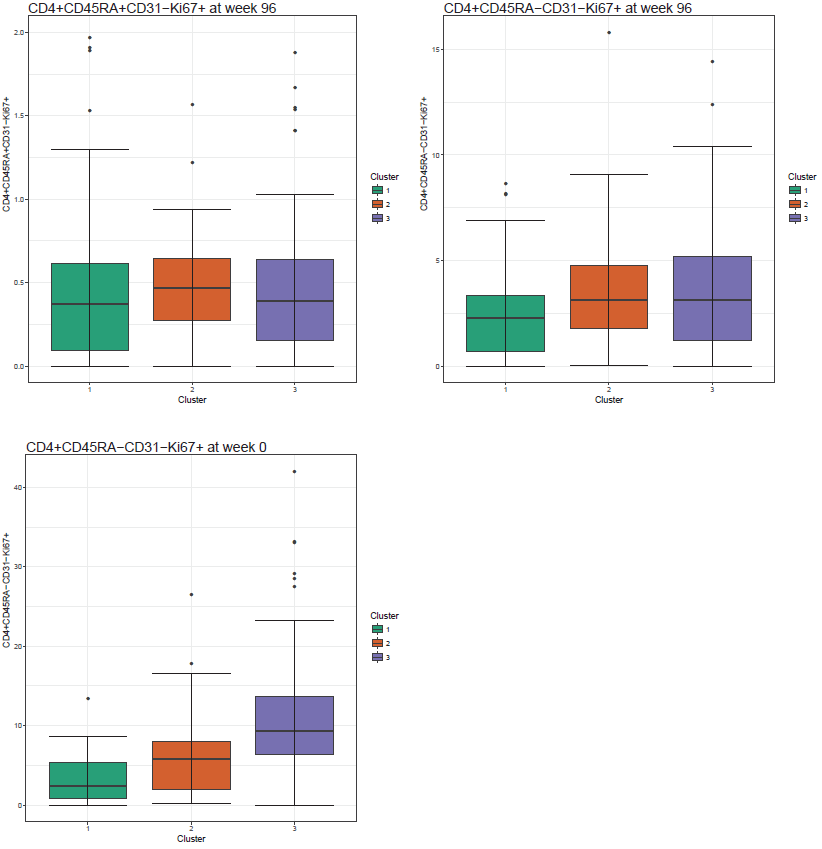


**Inflammation markers**

**
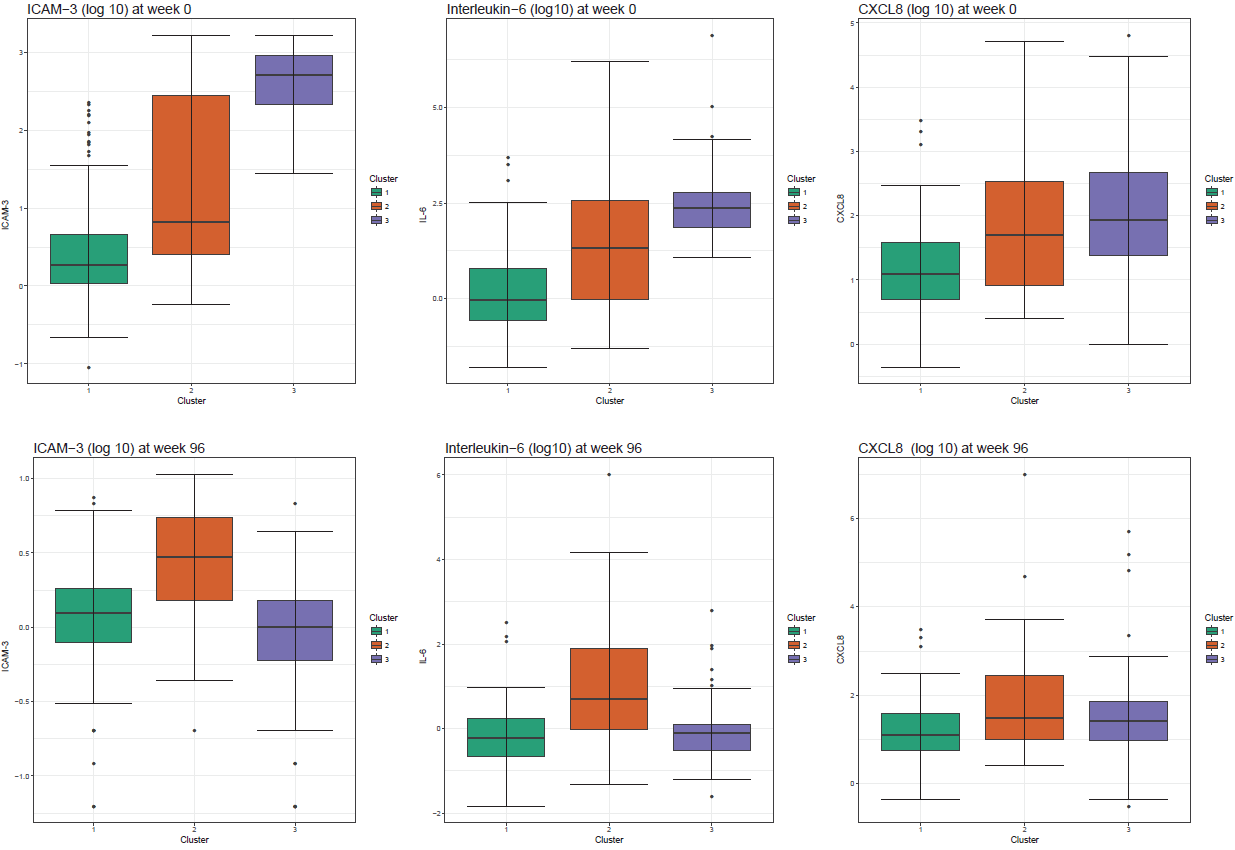
**

**
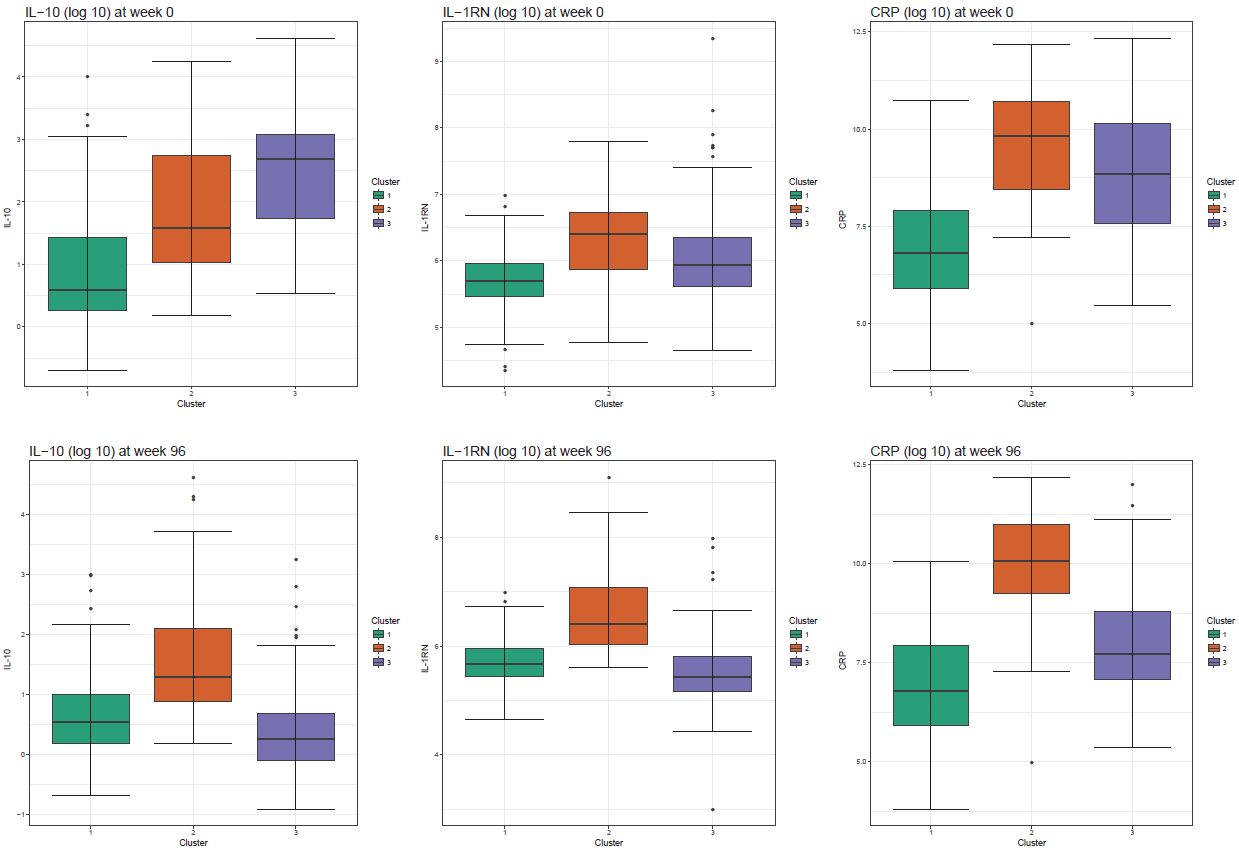
**

**
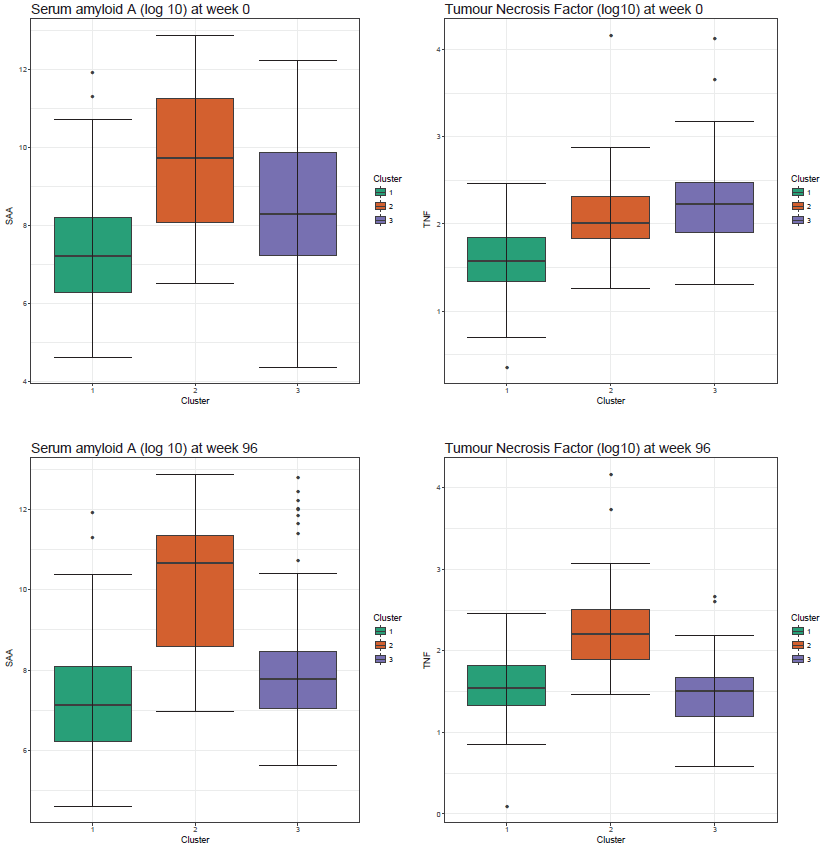
**

**Cardiovascular injury markers**


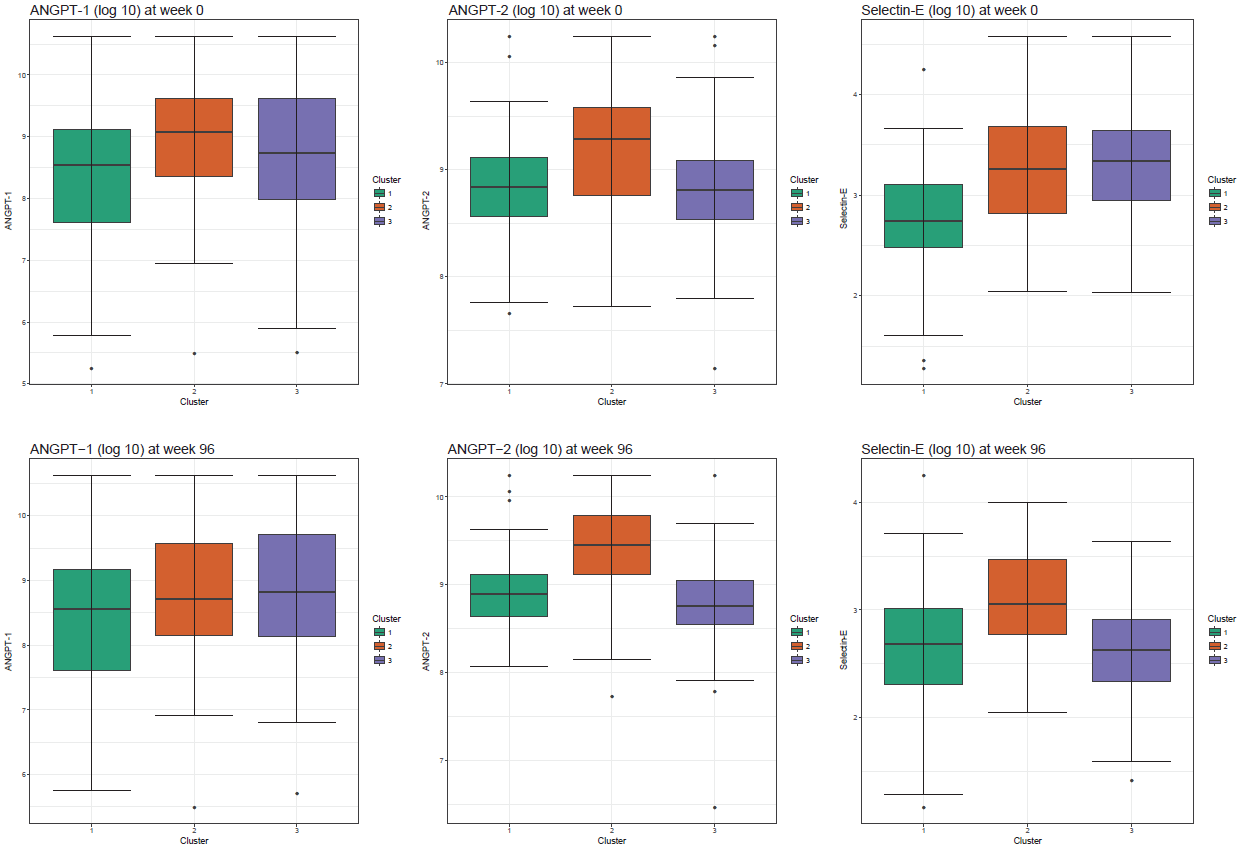


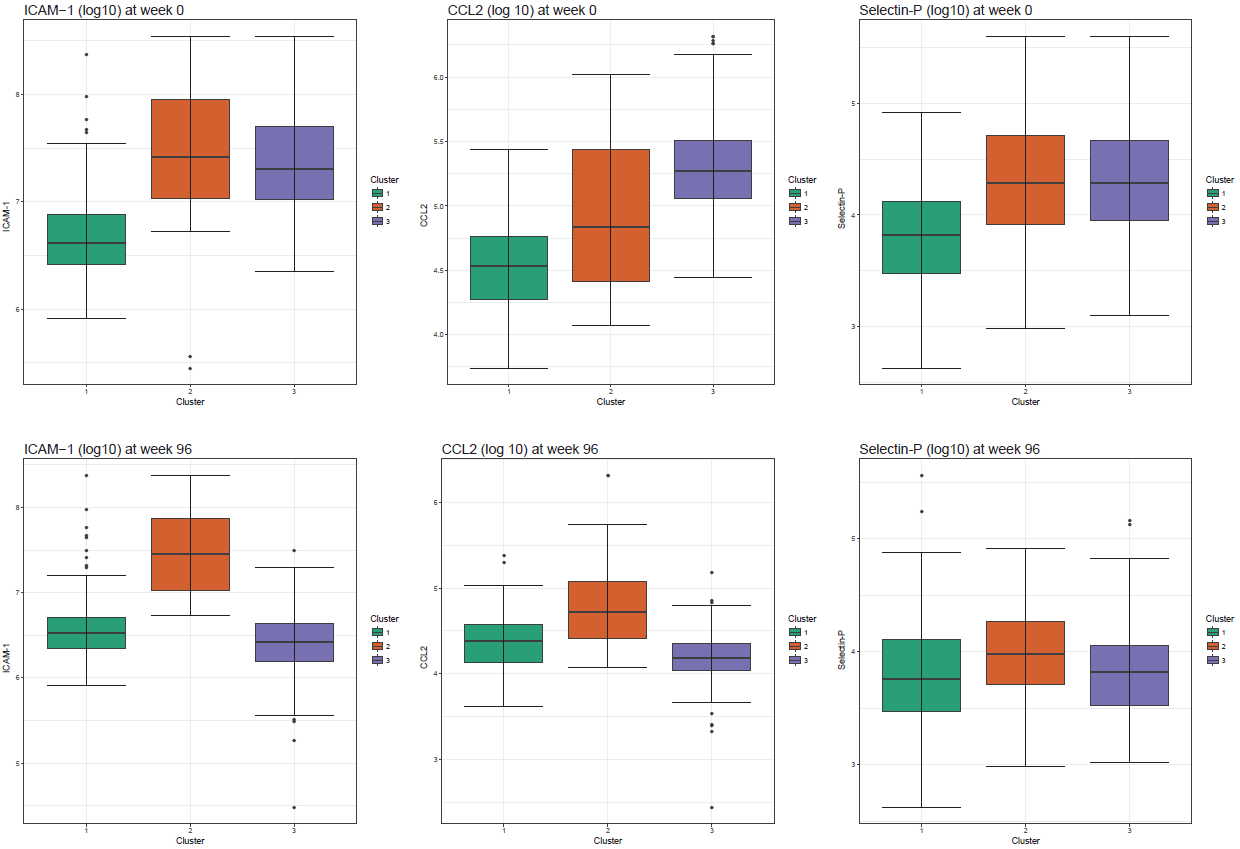


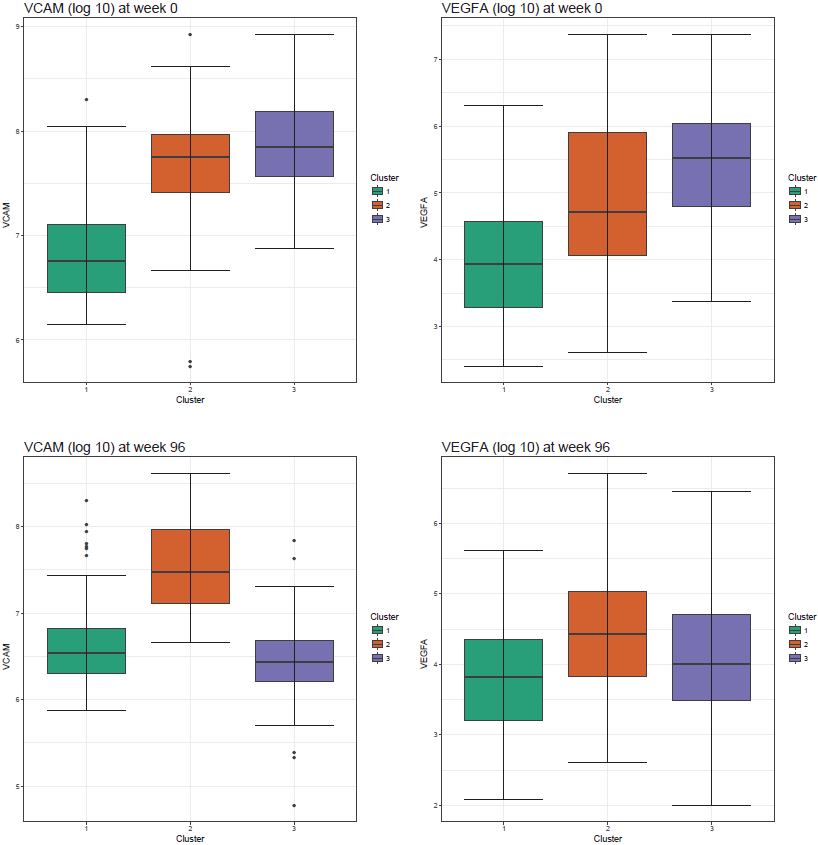


**Disordered thrombogenesis markers**


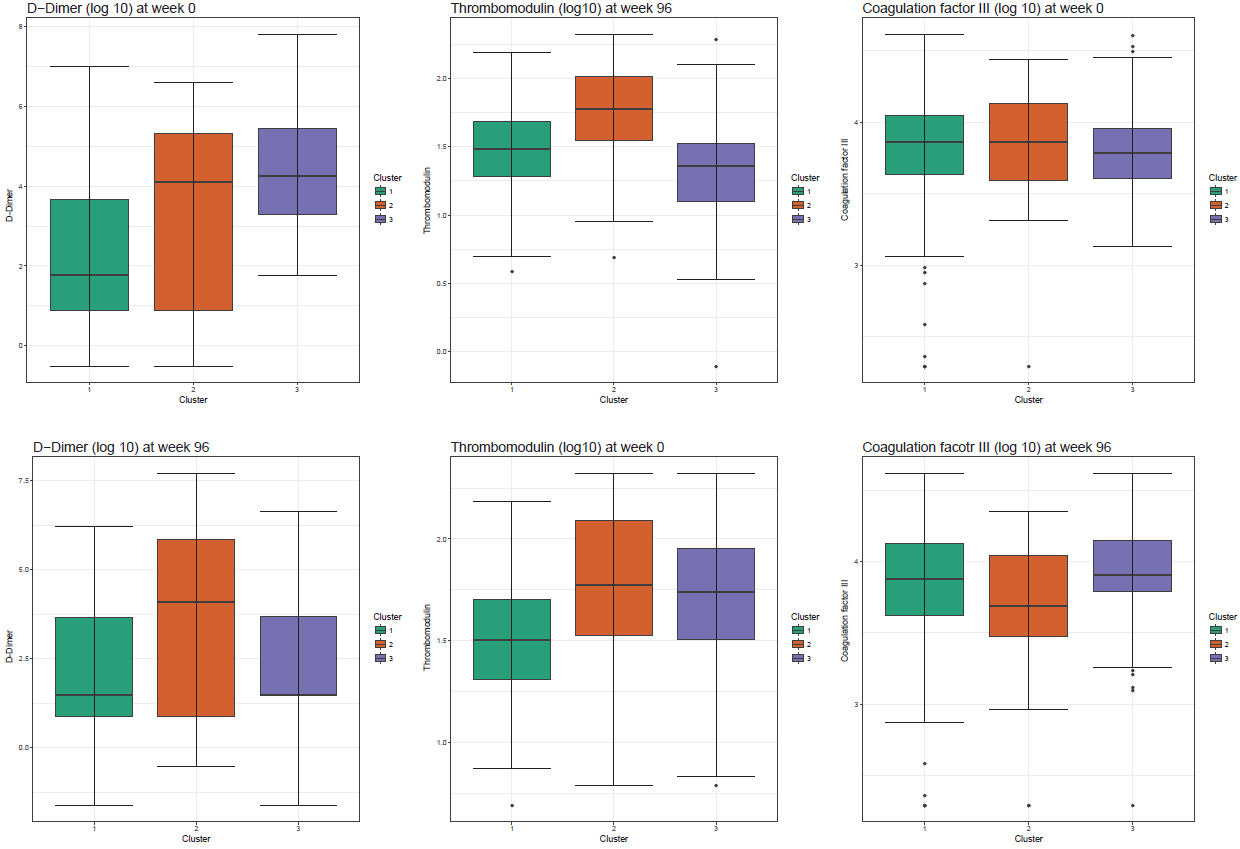


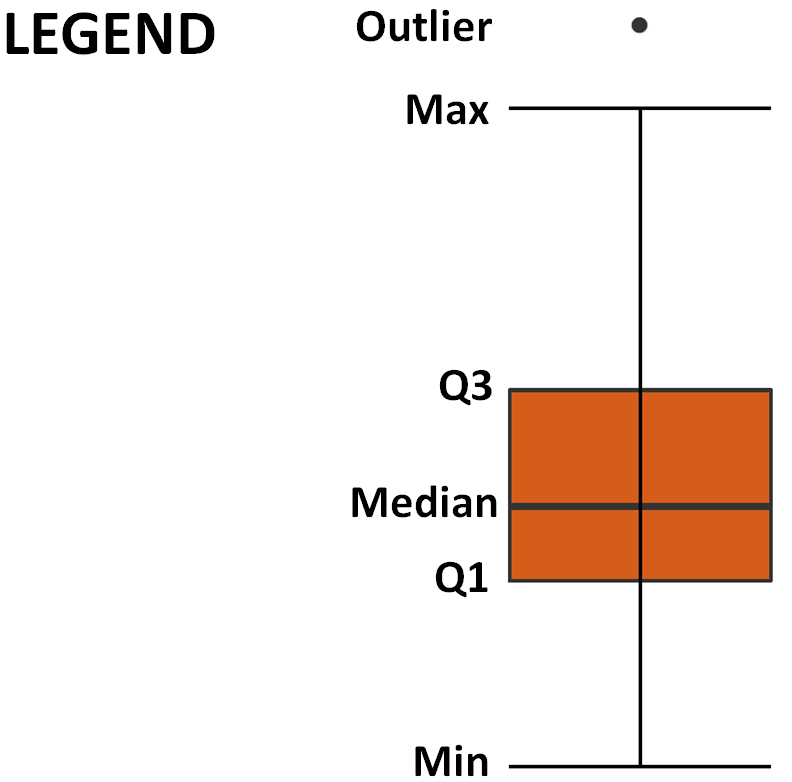


References

1. Fitzgerald F, Harris K, Doyle R, Alber D, Klein N. Evidence that Microbial translocation occurs in HIV-infected children in the United Kingdom. AIDS Res Human Retroviruses **2013**.

2. Caporaso JG, Kuczynski J, Stombaugh J, et al. QIIME allows analysis of high-throughput community sequencing data. Nat Methods **2010**; 7:335-6.

3. Magoc T, Salzberg SL. FLASH: fast length adjustment of short reads to improve genome assemblies. Bioinformatics **2011**; 27:2957-63.

4. DeSantis TZ, Hugenholtz P, Larsen N, et al. Greengenes, a chimera-checked 16S rRNA gene database and workbench compatible with ARB. Appl Environ Microbiol **2006**; 72:5069-72.

5. Freeman JV, Cole TJ, Chinn S, Jones PR, White EM, Preece MA. Cross sectional stature and weight reference curves for the UK, 1990. Arch Dis Child **1995**; 73:17-24.

6. World Health Organization. Global Database on Child Growth and Malnutrition. **2012**.

7. Huenecke S, Behl M, Fadler C, et al. Age-matched lymphocyte subpopulation reference values in childhood and adolescence: application of exponential regression analysis. Eur J Haematol **2008**; 80:532-9.

8. Hulstaert F, Hannet I, Deneys V, et al. Age-related changes in human blood lymphocyte subpopulations. II. Varying kinetics of percentage and absolute count measurements. Clin Immunol Immunopathol **1994**; 70:152-8.

9. Jiang W, Lederman MM, Hunt P, et al. Plasma levels of bacterial DNA correlate with immune activation and the magnitude of immune restoration in persons with antiretroviral-treated HIV infection. J Infect Dis **2009**; 199:1177-85.

10. BenMarzouk-Hidalgo OJ, Torres-Cornejo A, Gutierrez-Valencia A, Ruiz-Valderas R, Viciana P, Lopez-Cortes LF. Differential effects of viremia and microbial translocation on immune activation in HIV-infected patients throughout ritonavir-boosted darunavir monotherapy. Medicine (Baltimore) **2015**; 94:e781.

11. Abad-Fernandez M, Vallejo A, Hernandez-Novoa B, et al. Correlation between different methods to measure microbial translocation and its association with immune activation in long-term suppressed HIV-1-infected individuals. J Acquir Immune Defic Syndr **2013**; 64:149-53.

12. Sandler NG, Douek DC. Microbial translocation in HIV infection: causes, consequences and treatment opportunities. Nat Rev Microbiol **2012**; 10:655-66.

13. Pilakka-Kanthikeel S, Huang S, Fenton T, Borkowsky W, Cunningham CK, Pahwa S. Increased Gut Microbial Translocation in HIV-infected Children Persists in Virologic Responders and Virologic Failures After Antiretroviral Therapy. Pediatr Infect Dis J **2012**; 31:583-91.

14. Ellwood M, Nomura M. Deletion of a ribosomal ribonucleic acid operon in Escherichia coli. J bacteriol **1980**; 143:1077-80.

15. Klappenbach JA, Dunbar JM, Schmidt TM. rRNA operon copy number reflects ecological strategies of bacteria. Appl Environ Microbiol **2000**; 66:1328-33.

16. URI Genomics and Sequencing Center. Calculator for determining the number of copies of a template. Available at: <http://cels.uri.edu/gsc/cndna.html>. 2016.

17. Chevalier MF, Petitjean G, Dunyach-Remy C, et al. The Th17/Treg ratio, IL-1RA and sCD14 levels in primary HIV infection predict the T-cell activation set point in the absence of systemic microbial translocation. PLoS Pathog **2013**; 9:e1003453.

18. Kramski M, Gaeguta AJ, Lichtfuss GF, et al. Novel sensitive real-time PCR for quantification of bacterial 16S rRNA genes in plasma of HIV-infected patients as a marker for microbial translocation. J Clin Microbiol **2011**; 49:3691-3.

19. Wittkop L, Bitard J, Lazaro E, et al. Effect of cytomegalovirus-induced immune response, self antigen-induced immune response, and microbial translocation on chronic immune activation in successfully treated HIV type 1-infected patients: the ANRS CO3 Aquitaine Cohort. J Infect Dis **2013**; 207:622-7.

20. Madrid L, Noguera-Julian A, Falcon-Neyra L, et al. Microbial translocation and T cell activation are not associated in chronic HIV-infected children. AIDS **2014**; 28:1989-92.

21. Sabet NS, Subramaniam G, Navaratnam P, Sekaran SD. Simultaneous species identification and detection of methicillin resistance in staphylococci using triplex real-time PCR assay. Diagn Microbiol Infect Dis **2006**; 56:13-8.

22. Tann CJ, Nkurunziza P, Nakakeeto M, et al. Prevalence of bloodstream pathogens is higher in neonatal encephalopathy cases vs. controls using a novel panel of real-time PCR assays. PLoS One **2014**; 9:e97259.

23. Boutaga K, van Winkelhoff AJ, Vandenbroucke-Grauls CM, Savelkoul PH. Periodontal pathogens: a quantitative comparison of anaerobic culture and real-time PCR. FEMS Immunol Med Microbiol **2005**; 45:191-9.
